# Neuronal SKN-1B modulates nutritional signalling pathways and mitochondrial networks to control satiety

**DOI:** 10.1371/journal.pgen.1009358

**Published:** 2021-03-04

**Authors:** Nikolaos Tataridas-Pallas, Maximillian A. Thompson, Alexander Howard, Ian Brown, Marina Ezcurra, Ziyun Wu, Isabel Goncalves Silva, Christopher D. Saunter, Timo Kuerten, David Weinkove, T. Keith Blackwell, Jennifer M. A. Tullet

**Affiliations:** 1 School of Biosciences, University of Kent, Canterbury, United Kingdom; 2 Joslin Diabetes Center, One Joslin Place, Boston, Massachusetts, United States of America; 3 Magnitude Biosciences Ltd, NETPark Plexus, Sedgefield, United Kingdom; University of California San Francisco, UNITED STATES

## Abstract

The feeling of hunger or satiety results from integration of the sensory nervous system with other physiological and metabolic cues. This regulates food intake, maintains homeostasis and prevents disease. In *C*. *elegans*, chemosensory neurons sense food and relay information to the rest of the animal via hormones to control food-related behaviour and physiology. Here we identify a new component of this system, SKN-1B which acts as a central food-responsive node, ultimately controlling satiety and metabolic homeostasis. SKN-1B, an ortholog of mammalian NF-E2 related transcription factors (Nrfs), has previously been implicated with metabolism, respiration and the increased lifespan incurred by dietary restriction. Here we show that SKN-1B acts in two hypothalamus-like ASI neurons to sense food, communicate nutritional status to the organism, and control satiety and exploratory behaviours. This is achieved by SKN-1B modulating endocrine signalling pathways (IIS and TGF-β), and by promoting a robust mitochondrial network. Our data suggest a food-sensing and satiety role for mammalian Nrf proteins.

## Author summary

Deciding when and how much to eat is important for maintaining health and preventing disease. It requires an intricate molecular level of communication between our nervous, physiological, and metabolic systems. These signals stimulate food intake, and afterwards the feeling of satiety which makes us stop eating. We have studied these phenomena using the simple nematode worm *C*. *elegans* which has a fully mapped nervous system and quantifiable food-related behaviours. In *C*. *elegans*, chemosensory neurons sense food and communicate this to the rest of the animal via hormones to control food-related behaviour and associated physiological changes. Here we identify a new central node of this system, the *C*. *elegans* gene SKN-1B, which acts in two sensory neurons to sense food, communicate food-status to the rest of the worm, and control satiety and exploratory behaviours. It does this by altering hormonal signalling (Insulin and Transforming Growth Factor-β), and by promoting a strong mitochondrial network. The mammalian equivalents of SKN-1B are the NF-E2 related transcription factors (Nrfs), which have previously been implicated with metabolism and respiration. Our data suggest a new food-sensing and satiety role for mammalian Nrf proteins.

## Introduction

It is necessary for animals to correctly sense and adapt to food. Information on food cues is obtained via the sensory nervous system, integrated in the hypothalamus, and influences decisions about development, growth and behaviour [1,2]. These signals dictate appropriate food intake and regulate metabolic homeostasis, but are not well understood. In the nematode worm *C*. *elegans*, chemosensory neurons detect nutritional status, and relay this information to other tissues via hormones [3]. These hormones activate downstream intracellular mechanisms including the insulin/IGF-1-like signalling (IIS) and transforming growth factor-β (TGF-β) pathways which act to switch behaviour between roaming (looking for and consuming food), dwelling (consuming food) and quiescence (a sleep-like state linked to satiety) depending on nutritional availability [4–7]. Adaptation to food cues also requires physiological changes, and mitochondrial networks are modulated to maximise energy output [8]. Combined, these appropriate behavioural and physiological changes mean that food levels are correctly perceived, nutrient intake is regulated, and metabolic balance is maintained.

In mammals the NF-E2 related transcription factors (Nrfs) regulate a variety of processes. Nrf2 is known as a key, inducible, oxidative stress response factor but along with other Nrfs has also been implicated in proteostasis and metabolism [9]. *C*. *elegans*, has only one sequence and functional Nrf orthologue, SKN-1, but its outputs are thought likely to be distributed between all the mammalian Nrfs [9]. There are three *skn-1* isoforms (SKN-1A-C). SKN-1A and SKN-1C are expressed in the intestine and regulated, similarly to the Nrfs, at the level of cellular localisation [10,11]. In contrast, SKN-1B is expressed in two chemosensory neurons, the ASIs, which are thought to act as the worm’s hypothalamus, and is constitutively nuclear [3,10,12]. SKN-1B has been of particular interest with respect to metabolism and respiration, because its action in ASI can mediate the increased lifespan incurred by dietary restriction (DR) [12].

We further tested the role of SKN-1B in DR mediated longevity but found it to be non-essential. Instead, we identify SKN-1B to be deeply ingrained in food-detection and food-related behavioural responses. Specifically, we find that SKN-1B: regulates satiety in response to fasting and re-feeding; promotes exploration in fed conditions; and controls appropriate responses to fasting. Our data suggest that SKN-1B controls food-related behaviour both via modulating the key signalling pathways (TGF-β and insulin signalling), and physiologically through the control of mitochondrial networks. This places SKN-1B at the heart of food-responsive signalling pathways, where it acts to regulate satiety and control metabolic homeostasis. Our data suggest the possibility that Nrfs act to regulate food-sensing and satiety in humans.

## Results

### SKN-1B contributes to DR longevity, but is not necessarily essential

**Fig 1:**
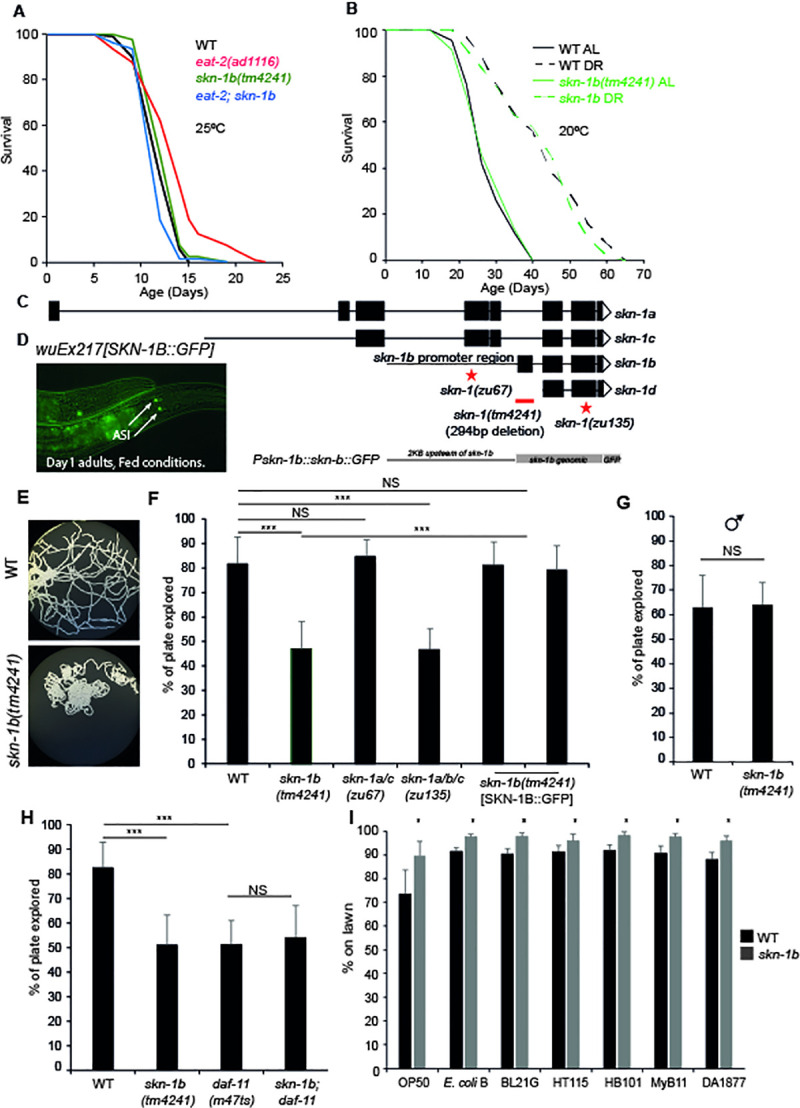
*skn-1b* is required for exploratory behaviour, but is not essential for DR longevity. **A)** Effect of *skn-1b* on *eat-2* lifespan. **B)** Survival of WT and *skn-1b* mutants in response to bacterial dilution as in [15]. For **A and B**: Representative experiments shown, individual trials summarised with Log-Rank analysis in S1 and S2 Tables. These DR protocols did not alter SKN-1B::GFP levels (S7B and S7C Fig). We use *eat-2* as a DR longevity model as suggested [59], but recent work shows that *eat-2* longevity also derives from reduced pathogenesis [60,61]. *C*. *elegans* derives nutrients from the bacteria and removing pathogenic components of bacteria will undoubtedly alter its nutritional profile but separating the two is challenging. **C)** Genetic locus of *skn-1* with isoforms, mutants and SKN-B::GFP specific transgene. *skn-1b* mRNA is not detectable in *tm4241* mutants but *skn-1a* and *c* mRNA levels are unchanged implying that this allele is *skn-1b* specific (S3A Fig). *skn-1b* mutants have normal brood sizes (S3B–S3E Fig). *skn-1(zu67)* and *skn-1(zu135)* encode point mutations leading to early stop codons and transcript degeneration via non-sense mediated decay. All *skn-1* isoforms have the same binding site, and all Nrfs can bind the same sequence, suggesting the likelihood of overlapping targets. **D)** *wuEx217* SKN-1B::GFP is expressed in ASI neurons. The SKN-1B::GFP translational reporter confirmed that SKN-1B::GFP can be expressed independently from other SKN-1 isoforms, that *skn-1b* is expressed solely in the ASIs. This expression pattern was confirmed with an endogenous Scarlet::SKN-1B reporter (S3F Fig). SKN-1B::GFP expression varies at different ages (S3G Fig). ASIs confirmed by DiI staining and SKN-1B::GFP was rarely observed in additional neurons (S3H Fig). **E)** Agar plates showing exploration of a single worm over 16hrs. Assay and controls shown (S4A and S4B Fig). **F-H)** Quantification of exploration. Mean plate coverage of n>11 worms per group ± st. dev. One representative experiment of 3 biological replicates shown. In **F)** SKN-1B was rescued using the *ukcEx15* and *ukcEx16* transgenes. In **G)** a 2hr period was used to allow quantification of hyperactive male exploration. **I)** Quantification of worms on different small bacterial lawns. Assay (S5A Fig). Each bar represents a mean of 3 biological replicates ± st. dev. For **F-I)** Two-tailed *t*-test *p<0.05, **p< 0.001, ***p<0.0001, NS not significant.

SKN-1 is a well characterised promoter of longevity: Mutants lacking all *skn-1* isoforms are short lived and mild overexpression of SKN-1 extends lifespan [9,13]. In particular, expression of *skn-1b* in the ASI neurons can mediate the extension in lifespan incurred by a food dilution DR protocol, suggesting that SKN-1B might be a general and essential mediator of DR (Bishop and Guarente, 2007). Multiple *C*. *elegans* DR protocols exist, some of which have different underlying genetic requirements [14], so we explored the specific *skn-1b* requirement for these other forms of DR. The weaker, ~20% lifespan extension observed in *eat-2* mutants required *skn-1b* (Fig 1A and S1 Table). However, an alternative food dilution protocol that extends WT lifespan more dramatically ~40–60%, and is dependent on *skn-1* [15], proved independent of *skn-1b* (Fig 1B and S2 Table). We conclude that although *skn-1b* contributes to DR mediated longevity under some conditions, it is not necessarily essential (Fig 1A and 1B and S3 Table)[12]. Like DR, reduced IIS (rIIS) extends lifespan in many species and *skn-1* is known to be an important mediator of this [10,16]. However, *skn-1b* was not required for the long life of *daf-2* mutants, suggesting either redundancy among isoforms or a requirement for other isoforms in particular (S1A–S1F and S2 Figs and S4 and S5 Tables). Neither did we observe any requirement of *skn-1b* for WT lifespan (S1 and S2 Figs and S4 and S5 Tables). In summary, *skn-1b* does not contribute to longevity under normal or rIIS conditions, but does contribute to the lifespan incurred by specific DR conditions.

### skn-1b acts to promote food-related exploratory behaviour

Sensory input via the ASIs affects *C*. *elegans’* three main food-related locomotory behaviours (roaming, dwelling and quiescence) [3,17]. Given that SKN-1B is implicated in DR longevity we explored the role of *skn-1b* in behaviour using a *skn-1b*-specific allele *(tm4241)* (Figs 1C and S3A–S3E). To gain an overview of food-related behavioural patterns, we quantified the ability of *skn-1b* mutants to “explore” a continuous bacterial lawn during a 16hr period compared to WT, an assay shown to correlate with classical roaming and dwelling assays [18] (S4A Fig). During this period, WTs explored ~80% of the lawn, but *skn-1b* mutants only explored ~45% suggesting that *skn-1b* mutants’ exhibit reduced exploratory behaviour (Fig 1E and 1F). We observed similar behaviour in *skn-1(zu135)* mutants which lack all *skn-1* isoforms, but not in *skn-1(zu67)* mutants which are thought to lack only *skn-1a* and *c* (Fig 1C–1F). Furthermore, rescuing *skn-1b* mutants with a SKN-1B::GFP specific transgene, which drives *skn-1b* expression from its own promoter specifically in the ASIs, fully restored exploratory behaviour to WT levels (Figs 1D, 1F and S3F).

As some *skn-1* isoforms are important for normal embryogenesis [19], it is possible that the *skn-1b* requirement for normal exploration could be due to disrupted ASI development. However, *skn-1* RNAi from the post-embryonic L1 or L4 stage was sufficient to decrease exploration, indicating that this phenotype is not due to a *skn-1b*-related embryonic development defect (S4C–S4E Fig). *skn-1b* mutants also performed as well as WT in an assay of thrashing behaviour indicating that their movement was not generally impaired (S4F Fig). We also explored behavioural differences in male *C*. *elegans* that have evolved to balance the competing needs of reproduction *versus* foraging. For instance, in the absence of hermaphrodites, males increase exploratory behaviour to search for mates [20,21]. However, we found that both WT and *skn-1b* males explored to the same hyperactive degree (Fig 1G). Thus, *skn-1b* promotion of motility appears to support foraging rather than mate location. Together, we conclude that adult expression of *skn-1b* in ASIs contributes to normal exploratory behaviour.

The ASI neurons consist of cell bodies that reside anterior to the large bulb of the pharynx, with projections reaching forward to the amphid openings (the worm’s nose) [3]. At the amphid openings, the ASIs express transmembrane receptor-type guanylate cyclases such as *daf-11* that relay environmental cues to the cell body [22]. *daf-11* mutants have sensory defects and fail to chemotax towards a number of attractants including NaCl and diacyl as well as being required for normal dauer entry and exit [22]. To explore the relationship between *skn-1b* and *daf-11* we tested their epistatic relationship in relation to behaviour. Similarly to *skn-1b* mutants, we observed an exploratory defect in *daf-11* mutants (Fig 1H) and notably, a *skn-1b; daf-11* double mutant did not exhibit a greater reduction in exploration (Fig 1H). The lack of an additive effect of these two mutations suggests that *daf-11* and *skn-1b* act in the same pathway to influence behaviour.

In exploration assays *C*. *elegans* are cultured on a continuous lawn of *E*. *coli*. As *skn-1b* mutants explore less, we reasoned that they may spend less time away from food than WTs. To test this, we provided the worms with a small lawn of OP50 bacteria in the centre of an otherwise empty plate, and counted the number of worms on and off the bacteria (S5A Fig). Whilst at any given time approximately 25% of WT worms are off a standard OP50 lawn, at the same point all *skn-1b* mutants remained on the lawn (Fig 1I). Similar mild avoidance of lawns in WT but not *skn-1b* mutants was seen for other bacteria, including another four *E*. *coli* strains *(E*. *coli* B, BL21G, HT115 and HB101*)*, *Comamonas aquatica* (DA1877) and a *Pseudomonas sp*. (MyB11) (Fig 1I). However, when WT worms are fed *B*. *subtilis* (PY79) the proportion on the lawn increases compared to OP50 whereas that of *skn-1b* mutants remains the same (S5B Fig). Similarly, no differences in lawn avoidance were seen on *E*. *coli* W3110 or MG1655 (S5B Fig). As almost all *skn-1b* mutants are present on an OP50 lawn, it implies that they are behaving in a satiated manner. We also tested whether *skn-1b* might contribute to a pathogen avoidance response and examined food avoidance behaviour of WT and *skn-1b* mutants fed pathogenic *Pseudomonas aeruginosa*. However, both WT and *skn-1b* mutants avoided the pathogen to a similar extent indicating that *skn-1b* is not involved in pathogen avoidance behaviour (S5C Fig). Overall, this indicates that *skn-1b* acts to sense food types rather than pathogenicity and subsequently controls behaviour.

### skn-1b regulates behaviour in response to fasting

**Fig 2:**
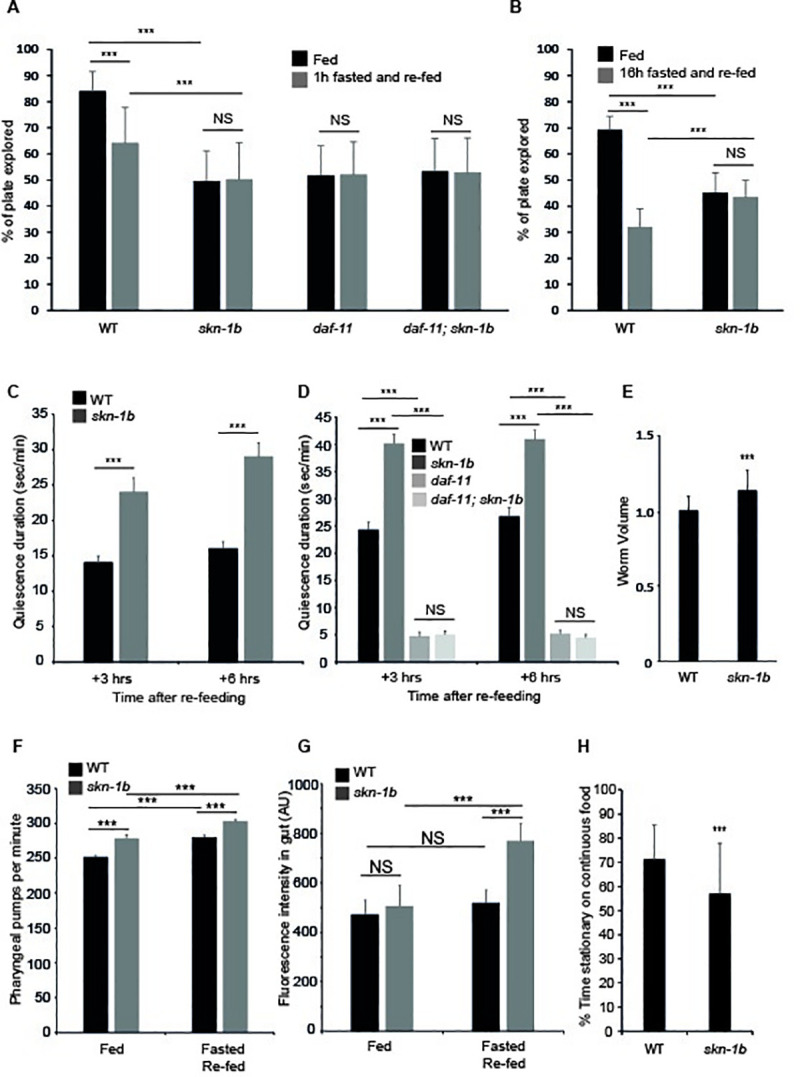
SKN-1B regulates satiety quiescence. **A)** Quantification of exploration in fed *vs* fasted/re-fed conditions, worms fasted for 1hr. Mean plate coverage of n>35 individual worms per group ± st. dev., 3 combined experiments shown. **B)** Quantification of exploration in fed *vs* fasted/re-fed conditions, worms fasted for 16hrs. Mean plate coverage of n>7 worms per group ± st. dev., one representative experiment of 3 trials shown. **C and D)** Time spent in quiescence after fasting/re-feeding. Each bar represents a mean of 3 biological replicates ± SEM, n>40 worms per group. **E)** Worm volume. Each bar represents a mean of 3 biological replicates, ± st. dev., n>63 worms per group. **F)** Pharyngeal pumping rate. Each bar represents a mean of 3 biological replicates, ± st. dev., n = 7 worms per group. **G)** Effect of *skn-1b* on intake of fluorescently labelled OP50. Each bar represents a mean of 3 biological replicates, ± st. dev., n>42 worms per group. **H)** Automated measure of movement on a continuous lawn of OP50. Each bar represents a mean of 3 biological replicates, ± st. dev., n>54 worms per group. For **A-H**) Two-tailed *t*-test *p<0.05, **p< 0.001, ***p<0.0001, NS not significant.

Exploration allows worms to seek and locate food [4]. When re-fed after a period of fasting, exploration is reduced and worms ‘dwell’ to increase their food consumption and refuel their energy stores, they then enter satiety quiescence [4,5]. These responses are regulated by the ASIs and hormones, so we investigated the contribution of *skn-1b*. We fasted WT and *skn-1b* mutants for 1hr and quantified their behaviour upon re-feeding. Whilst WT worms exhibited the expected reduction in exploration under these conditions, *skn-1b* mutants did not (Fig 2A). We also fasted WT and *skn-1b* mutants for 16hrs, and examined their exploration following re-feeding. We found that while this more extreme fasting protocol caused a marked decrease in WT activity compared to 1hr fasting, it had no effect on *skn-1b* mutants (Fig 2A and 2B). This demonstrates that *skn-1b* is required for behavioural control in response to fasting and re-feeding.

As *daf-11* mutants also exhibit decreased exploration, (Figs 1H and 2A), we tested whether *daf-11* was required for *skn-1b* mediated behavioural changes in response to fasting and re-feeding. We found that *daf-11* worms, like *skn-1b* mutants, do not respond to fasting and re-feeding, and that the combined effect of *daf-11; skn-1b* mutation was non-additive (Fig 2A). That both DAF-11 and SKN-1B are required for worms to modulate their exploratory behaviour in response to fasting and re-feeding provides further evidence that these two proteins act in concert.

The decreased exploration observed in response to fasting and re-feeding can be attributed either to increased time spent dwelling, or in quiescence [4,5]. When dwelling, pharyngeal pumping is normal and *C*. *elegans* makes minimal back and forward sinusoidal movement [3]. In contrast, quiescent worms do not pump or move at all [5,6]. As *skn-1b* mutant’s exhibit reduced exploration we asked whether they also differ in these other behaviours. After fasting, WT worms are quiescent for a longer period during the 3-6hrs after re-feeding, making this the best time to measure satiety quiescence [5,6]. We found that at both 3 and 6hrs after re-feeding, *skn-1b* mutants spent longer in a quiescent state than WT worms (Fig 2C). Similar numbers of WT and *skn-1b* mutants quiesce under these conditions (S6A Fig). Together, this suggests that SKN-1B acts to suppress satiety-induced quiescence promoting exit from, but not entry into quiescence.

One factor controlling satiety induced quiescence is *daf-11*, and *daf-11* mutants cannot quiesce [5]. To test the relationship between *skn-1b* and *daf-11* in this regard we measured quiescence in *daf-11; skn-1b* double mutants. Although *daf-11* mutation did slightly reduce the % of *skn-1b* mutants entering quiescence by ~20%, we found that *daf-11* completely suppressed the long quiescence duration of *skn-1b* mutants (Figs 2D and S6B). This suggests that SKN-1B and DAF-11 have opposing roles in controlling satiety induced quiescence, but that SKN-1B requires functional DAF-11 to act as a molecular switch.

Quiescence is linked to satiety in mammals, and quiescent *C*. *elegans* do not pump food into their gut, so these data could imply that *skn-1b* mutants eat less compared to WT. We observed that *skn-1b* mutants are approximately 10% larger than WT (Fig 2E). In addition to the time spent on food, the amount of food that a worm eats can be determined by the efficiency and rate of pharyngeal pumping and the amount of time that it spends pumping [5]. To test this, we compared pumping rate in fed WT and *skn-1b* mutants and observed a modest but statistically significant increase in the latter (Fig 2F). This suggested that *skn-1b* mutants might ingest more *E*. *coli* than WT animals. To explore this further we examined food intake by quantifying uptake of fluorescently labelled OP50. If worms were fed mcherry labelled OP50 continuously (fed conditions), the guts of WT and *skn-1b* mutants contained similar amounts of bacteria (Fig 2G). However, in response to fasting and re-feeding *skn-1b* mutants accumulated more OP50 than WT, corresponding to a further increase in pumping rate under these conditions (Fig 2F and 2G). Together this suggests that *skn-1b* mutation alters feeding and quiescence associated parameters.

As *skn-1b* mutants exhibit increased satiety induced quiescence, it was possible that they also quiesce more in the presence of food. To test this we measured quiescence in fed conditions using an automated tracking system that detects the time *C*. *elegans* spends stationary *vs* moving. We assumed that stationary worms are quiescing as both roaming and dwelling involve movement. We found that in fed conditions, *skn-1b* mutants spent less time stationary than WTs, indicating that they quiesce less in fed conditions (Fig 2H). This contrasts with our data in fasted and re-fed conditions, and indicates that *skn-1b* is specifically required for satiety induced quiescence.

### Neuronal SKN-1B expression responds to specific food cues

**Fig 3:**
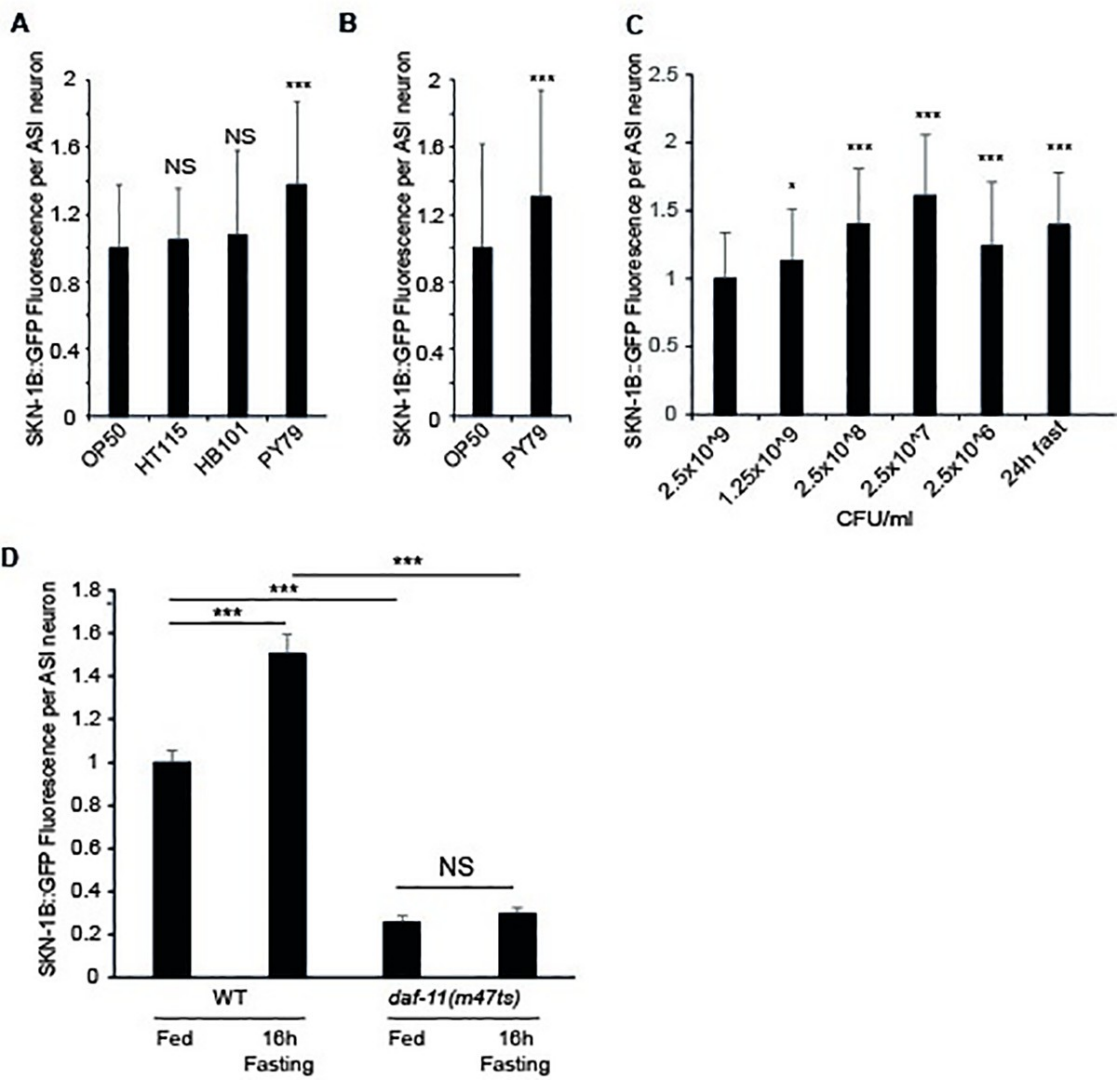
SKN-1B::GFP levels respond to nutritional cues and require *daf-11*. **A-D)** Quantitative fluorescence microscopy of SKN-1B::GFP in response to: **A)** different bacterial strains, **B)** being switched to PY79 at the L4 stage, **C)** 24hrs bacterial dilution [12], or **C)** 16hrs fasting. For **D)** a combination of *daf-11* mutation and fasting was used. Similar results to those in **C**) had previously been observed using a SKN-1B/C::GFP transgene [12]. **A-D)** Imaged at 1 day adults, each bar is the mean of 3 biological replicates ± st. dev. Two-tailed *t*-test *p<0.05, ***p<0.0001, NS not significant.

ASI neurons detect the worm’s environment, including food cues [3]. As *skn-1b* mediates food-related behaviours (Figs 1 and 2), and can contribute to DR lifespan extension (Fig 1A and S3 Table) [12] we examined SKN-1B expression levels in response to dietary changes. Laboratory *C*. *elegans* are fed a homogeneous diet of *E*. *coli* OP50, but can thrive on other bacterial lawns [23]. To test whether SKN-1B levels also respond to changes in food type we measured SKN-1B::GFP levels in the ASIs in *C*. *elegans* grown on different bacterial strains compared to OP50. SKN-1B::GFP levels were not altered when worms were cultured on *E*. *coli* HT115 or HB101, but increased in response to *Bacillus subtilis* (PY79) or *P*. *aeruginosa* (PA14) (Figs 3A, 3B and S7A). This induction of expression was rapid, e.g. occurring after 16hrs on *B*. *subtilis* (Fig 3B) and suggests that neuronal SKN-1B::GFP expression increases specifically and rapidly in response to different bacterial diets.

As *skn-1b* contributes to DR longevity we also examined the effect of DR on SKN-1B::GFP levels. We found that diluting bacteria in liquid culture increased ASI expression of SKN-1B::GFP (Fig 3C) [12], and that a similar increase was also observed when worms were fasted for 24hrs (Fig 3C). However, the alternative bacterial dilution DR protocol [15] nor the *eat-2* mutation had any effect on SKN-1B::GFP levels (S7B and S7C Fig). These data suggest that neuronal SKN-1B levels respond selectively to the amount of food available.

As behavioural effects of *skn-1b* and *daf-11* showed an interaction, we examined their relationship in respect to SKN-1B levels. Interestingly, without functional *daf-11*, SKN-1B::GFP levels were both significantly reduced, and could no longer increase in response to a 24hr fast (Fig 3D). Thus, SKN-1B requires functional *daf-11* to respond to the environment. Together with our behavioural analysis, and given the ASI expression patterns of DAF-11 (amphid opening) and SKN-1B (nucleus), this implies an epistatic relationship for these molecules, linking the external environment to SKN-1B levels and subsequent behaviours.

### SKN-1B requires TGF-β signalling to specify satiety-induced quiescence

**Fig 4:**
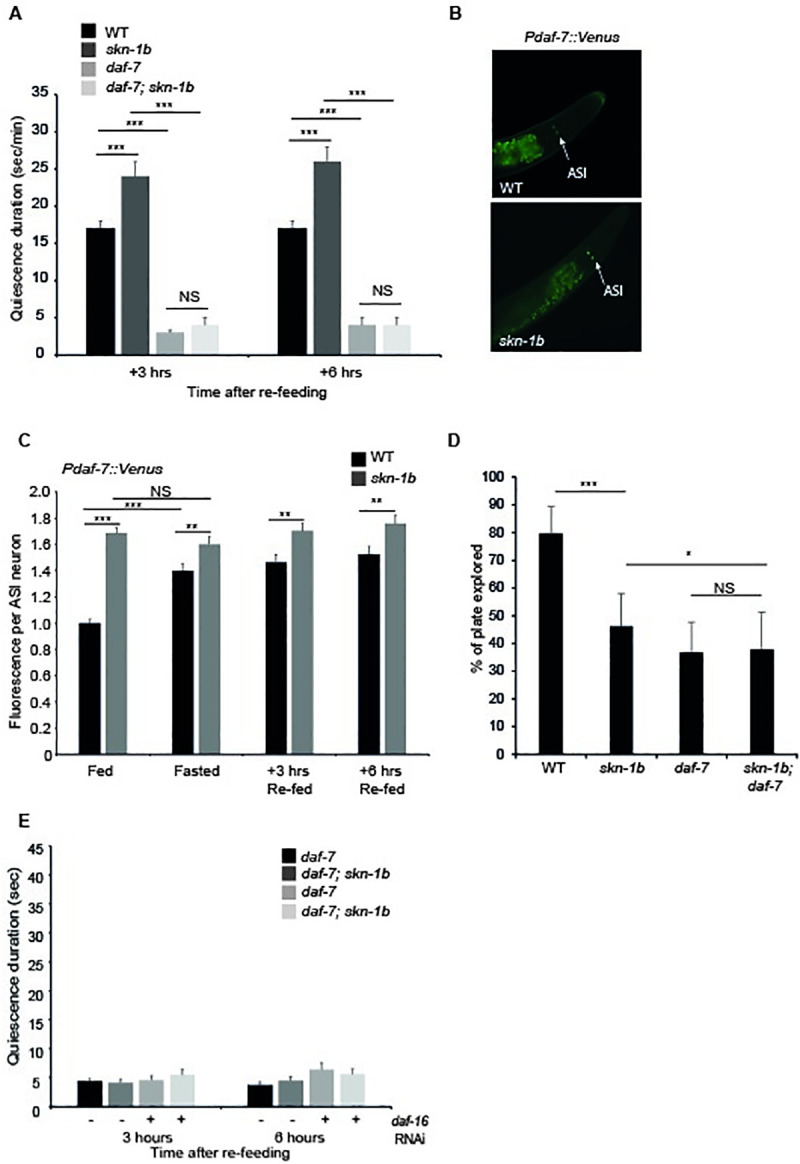
SKN-1B modulates TGF-β signalling and controls satiety. **A)** Time spent in quiescence after fasting and re-feeding. Each bar represents a mean of 3 biological replicates, ± SEM, n>9 worms per group. **B and C)** Fluorescence expression pattern, 20x magnification **(B),** and levels **(C),** of *Pdaf-7*::*GFP* in ASIs responds to *skn-1b* mutation and food cues. In **(C)** each bar represents a mean of 3 biological replicates ± st. dev., n>230 worms per group. NS difference was found between WT samples in fasted *vs* re-fed conditions and NS difference was found between *skn-1b* samples at any point. This regulation of *daf-7* is unlikely to be direct as there is no SKN-1 binding site within 3Kb of its transcriptional start site. **D)** Quantification of exploration. Each bar is a mean of 5 biological replicates, n>44 worms per group ± st. dev. All trials shown in S8A–S8F Fig. **E)** Time spent in quiescence after fasting and re-feeding. Each bar represents a mean of 3 biological replicates, ± SEM, n>10 worms per group. For **A, C, D** and **E**: Two-tailed *t*-test *p<0.05, **p< 0.001, ***p<0.0001, NS not significant.

Our data show that *skn-1b* is required in the ASIs to regulate food-related behaviours (Figs 1D–1I and 2A–2C). One way that ASIs act is by relaying chemosensory information to the rest of the worm via secretion of neuropeptides [3]. One of these, DAF-7, is the ligand of the canonical TGF-β signalling pathway, but its upstream regulators are not known [24]. ASIs secrete DAF-7 under environmental conditions favourable for growth and reproduction, and DAF-7 expression is highest when worms show high levels of quiescence [24,25]. In addition, expression of *daf-7* in ASI has been shown to promote quiescence, whilst *daf-7* mutants do not undergo satiety quiescence [5,26]. As *skn-1b* mutants’ exhibit enhanced quiescence we reasoned that *daf-7* might be a contributing factor. To test this, we generated *daf-7*; *skn-1b* mutants and measured their ability to undergo quiescence in response to fasting and re-feeding. In agreement with published work, WT animals showed increased quiescence following re-feeding, but *daf-7* mutants did not (Fig 4A [5,26]). As before, *skn-1b* mutants spent longer than WT in quiescence (Figs 2C and 4A), but this effect proved to be completely *daf-7* dependent (Figs 4A and S6C). In parallel we examined the expression of a *Pdaf-7*::*Venus* reporter in WT and *skn-1b* mutants. Similarly to *skn-1b*, *daf-7* is only expressed in ASI neurons but its expression increases in response to fasting and remains high for at least 6hrs, presumably supporting entrance into quiescence (Fig 4B and 4C). However, *skn-1b* mutants showed strongly elevated *Pdaf-7*::*Venus* expression in fed conditions, which barely altered in response to fasting or re-feeding (Fig 4B and 4C). Taken together, these data imply that SKN-1B inhibits satiety quiescence in response to fasting and re-feeding by suppressing *daf-7* expression and subsequently TGF-β signalling. *daf-7* mutants explore less than WT in fed conditions, and in this respect resemble *skn-1b* mutants (Fig 4D) [4,26]. To further investigate the behavioural epistasis relationship between *daf-7* and *skn-1b*, we examined the exploration of *daf-7*; *skn-1b* double mutants, but found that *daf-7* and *skn-1b* effects were non-additive (Figs 4D and S8).

### SKN-1B modulates IIS to alter food-responsive behaviour

**Fig 5:**
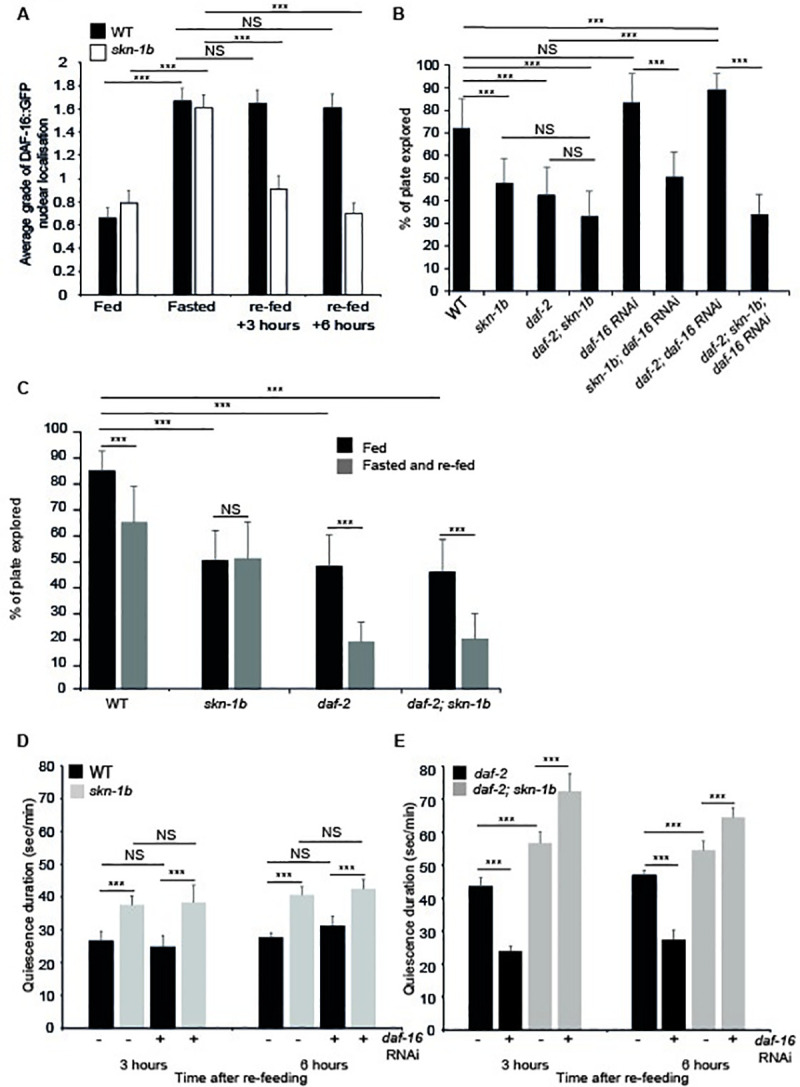
SKN-1B regulates IIS to control behaviour. **A)** Quantification of nuclear localisation, WT and *skn-1b* worms expressing *ges-1p*::*GFP*::*daf-16* [62], average grading shown. Grading system and total % of worms in each grade (S9 Fig). Combined data from 3 biological replicates shown ± SEM, n>48 worms per group. **B)** Quantification of exploration. One representative of 3 biological replicates shown ± st. dev., n>10 worms per group. **C)** Quantification of exploration in fed *vs* fasted and re-fed conditions. Worms fasted for 1hr. One representative of 3 biological replicates shown ± st. dev., n>35 worms per group. **B and C)** Similar findings were obtained using *daf-2(e1368)* (S11A and S11B Fig). **D and E)** Time spent in quiescence after fasting and re-feeding. Each bar represents a mean of 3 biological replicates ± SEM, total of n>36 worms per group. For **C-E)** Similar numbers of worms from each group were observed in quiescence (S6D–S6F Fig). For **A-E**: Two-tailed *t*-test *p<0.05, **p< 0.001, ***p<0.0001, NS = not significant.

*C*. *elegans* express and secrete ~40 insulin-like peptides (ILPs), at least some of which bind to the DAF-2 insulin/IGF-1-like receptor in multiple tissues [27]. rIIS leads to the de-phosphorylation and nuclear localisation of its downstream target the FOXO transcription factor DAF-16 [28,29]. Activation of DAF-16 has been implicated in a variety of phenotypes including behaviour, longevity, immunity and others–many of which are mediated by DAF-16 activity in the gut [4,6,7,30,31]. To test the impact of *skn-1b* on this pathway, we examined the cellular localisation of a gut-specific DAF-16a::GFP reporter in both WT and *skn-1b* mutants. In fed conditions, *skn-1b* did not affect DAF-16 nuclear localisation (Fig 5A), but fasting for 16hrs led to DAF-16a::GFP accumulation in both WT and *skn-1b* gut nuclei (Figs 5A and S9A–S9C). Strikingly worms lacking *skn-1b* could not maintain DAF-16::GFP in their gut nuclei after re-feeding, as WT worms do, reverting to WT levels of nuclear DAF-16::GFP within 3hrs of being returned to food (Figs 5A and S9A–S9C) [32,33]). Thus, *skn-1b* is required to maintain DAF-16 in the nucleus in response to fasting and re-feeding.

Some ILPs, have *skn-1* binding sites in their promoters, making direct regulation by SKN-1B possible. One of these is *ins-7* which is expressed in several neurons, including ASIs, and the gut [34,35]. We observed an increase in a *Pins-7*::*GFP* transcriptional reporter in both the neurons and gut of *skn-1b* mutant worms (S10A and S10B Fig). INS-7 is reported to be an agonist of DAF-2 in the gut while itself being transcribed downstream of rIIS, resulting a positive feedback loop which propagates and amplifies a downregulation of IIS in this tissue [34]. Increased expression of *ins-7* in the gut might therefore explain the reduced DAF-16 nuclear localisation we observe in *skn-1b* mutants in response to fasting and re-feeding (Fig 5A).

IIS is also implicated in food-related behaviour, and *daf-2(e1370)* mutants exhibit reduced exploration, similar to *skn-1b* mutants, dependent on *daf-16* (Fig 5B; [4,36]). To try to clarify the regulatory relationship between SKN-1B and IIS we examined the relationship between *skn-1b* and *daf-16* in our behavioural assays by knocking down *daf-16* mRNA using RNAi in WT, *daf-2(e1370)*, *skn-1b*, and double *daf-2(e1370); skn-1b* mutants. Knockdown of *daf-16* had no effect on the exploration of either WT or *skn-1b* mutants alone, but rescued the exploration deficiency of *daf-2* mutants back to WT levels (Fig 5B [4,6,7]). Surprisingly however, *daf-16* RNAi had no effect on the exploration of *daf-2; skn-1b* mutants (Fig 5B). We also examined the relationship between *daf-2* and *skn-1b* in response to food. With food, the reduced exploration of *daf-2* and *skn-1b* mutants was non-additive suggesting that they act in the same pathway (Fig 5B). However, *skn-1b* and *daf-2* mutants respond differently to fasting and re-feeding: *skn-1b* mutant behaviour is completely unresponsive; but *daf-2* mutants respond like WT, reducing their exploration upon re-feeding, a phenotype that seems independent of *skn-1b* (Figs 2B and 5C) [4–7]. As the class 1 allele *daf-2(e1370)* already exhibits very low exploratory behaviour it may be difficult to suppress further, so we also tested a ‘weaker’ class 2 allele *daf-2(e1368)* which exhibits a milder exploratory defect (S11A and S11B Fig) [37]. Similarly to our *e1370* results however, *daf-2(e1368)* and *skn-1b* exploratory defects were non-additive in both fed, and fasted and re-fed conditions (S11A and S11B Fig). These data could imply either that *skn-1b* acts upstream of *daf-2* to control exploration in response to fasting and re-feeding, or that *daf-2* acts independently of *skn-1b* to control this behaviour. Overall, our data suggest that for rIIS conditions DAF-16 acts to reduce exploration and SKN-1B acts to promote it.

Our data show that *skn-1b* impacts on DAF-16 regulation in response to fasting and re-feeding, and *skn-1b* mutants cannot maintain DAF-16 in gut nuclei under these conditions (Fig 5A). rIIS increases time spent in satiety quiescence dependent on DAF-16 [6,7]. Thus, we decided to explore whether *daf-16* contributes to the high levels of quiescence in our *skn-1b* mutants under rIIS conditions. We found that whilst *daf-16* RNAi had no effect on either WT or *skn-1b* mutant quiescence, *daf-2* mutation enhanced quiescence compared to WT, an effect suppressed by *daf-16* RNAi (Figs 5D, 5E, S6D and S6E). This supports the fact that *daf-16* is required for quiescence in the absence of IIS (Fig 5E) [6,7]. Addition of *skn-1b* mutation however, further increased *daf-2* quiescence, and this was not suppressed by *daf-16* RNAi (Figs 5E and S6E). Indeed, the quiescence of *daf-2; skn-1b; daf-16* RNAi treated animals was even higher than *daf-2; daf-16* RNAi (Fig 5E). That *daf-16* RNAi did not affect either WT or *skn-1b* mutant satiety, indicates that IIS must be reduced for this interaction to occur (Fig 5D and 5E). Overall, these data suggest that SKN-1B acts to maintain nuclear DAF-16, and in doing so allows DAF-16 to promote quiescence in response to rIIS. Together with our other data, these results imply that SKN-1B acts to modulate both TGF-β and IIS in response to food, allowing the outputs of these pathways to control behaviour, placing it as a new central node in ASI behavioural response pathways.

TGF-β and Insulin signalling also interact and increased nuclear localisation of DAF-16 is observed in *daf-7* mutants (Shaw et al 2007). Although *daf-7* mutants do not quiesce (Gallagher et al., 2013; You et al., 2008; Fig 4A), and *daf-7* fully suppresses *skn-1b* mutant quiescence (Fig 4A), we wondered if removal of DAF-16 had the potential to alter this relationship. To test whether IIS and TGF-β interact to control quiescence downstream of *skn-1b* we measured satiety induced quiescence in *daf-7* and *daf-7; skn-1b* mutants with and without *daf-16* RNAi. However, *daf-16* RNAi did not cause any changes in quiescence for either *daf-7* or *daf-7; skn-1b* mutants (Figs 4E and S6F). We conclude that IIS and TGF-β do not interact to control quiescence downstream of SKN-1B.

### SKN-1B controls behaviour by maintaining mitochondrial networks in muscle

Our data suggest that SKN-1B acts cell-non-autonomously to regulate behaviour. As food sensing and consumption is closely linked to physiological and metabolic homeostasis [8,38,39], this suggests that *skn-1b* dysregulation could cause physiological and metabolic disruption to the organism. *skn-1b* is required for normal behavioural responses to fasting (Figs 1E, 1F and 2A–2C), but *skn-1b* mutants are not actually starved (Fig 2D and 2E). Despite this, we noted that whilst a population of WT worms evenly distributes over a bacterial lawn, *skn-1b* mutants display a strong preference for the thicker outer edge “bordering” (S12A and S12B Fig). The edge of the lawn is considered to have reduced levels of O_2_ (~8%), and bordering has been associated with social behaviours, memory, temperature and starvation [40]. This suggests that *skn-1b* mutants exhibit signs of starvation despite being well fed. Given that *skn-1b* mutants are unable to appropriately perceive and respond to food cues, we explored whether the physiological state of *skn-1b* mutants differs from WT.

**Fig 6:**
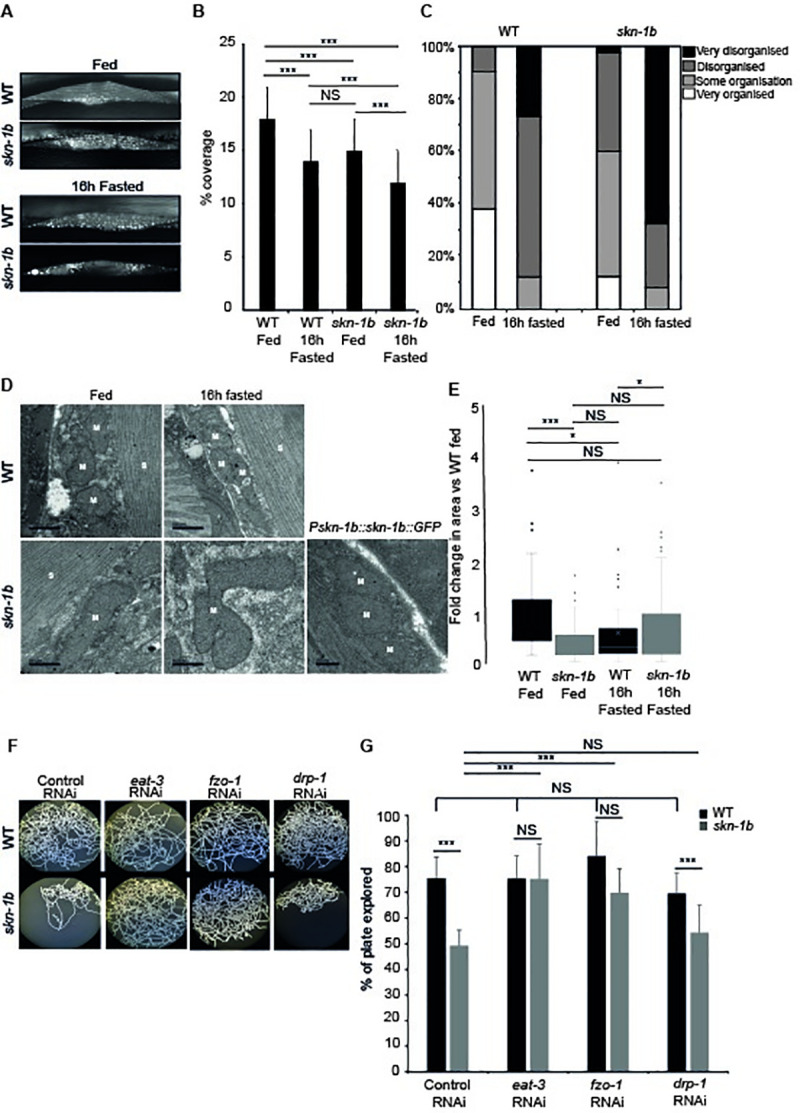
*skn-1b* contributes to mitochondrial network integrity. **A-C)** Expression and quantification of WT and *skn-1b* mutant worms expressing *myo-3*::*GFP(mit)*. This muscle specific reporter expresses an outer mitochondrial membrane protein and hence marks all mitochondria, delineating their shape. In **B and C)** Each bar represents a mean of 3 biological replicates ± SEM, n>62 day 1 adults worms per group. The qualitative scoring system used in **C)** is shown in S12C Fig. **D)** Longitudinal sections imaged by Transmission Electron Microscopy (TEM). M = mitochondria, S = sarcomere. Scale bar = 500nm. **E)** Quantification of TEM: Mitochondrial area compared to WT control. Each bar represents a mean of 2 biological replicates, n>47 images per group ± SEM. **F-G)** Effect of mitochondrial fission and fusion on mitochondrial networks and behaviour in WT and *skn-1b* mutants. Controls for effectiveness of RNAi (S14A and S14B Fig). For all graphs: Two-tailed *t*-tests *p<0.05, **p< 0.001, ***p<0.0001, NS not significant.

Mitochondria are dynamic organelles that change their network morphology, balancing their fission with fusion to maximise energy production [8,39,41]. In worms their morphology has been shown to change in response to starvation [42] as well as various DR protocols [39,43], and can be used to provide clues about an animal’s physiological state. In addition *skn-1* has previously been implicated in the maintenance of muscle mitochondrial networks, and anoxia-induced mitochondrial dynamics, raising the question of whether these phenomena might be mediated by *skn-1b* [44,45]. To explore the possibility that *skn-1b* impacts mitochondria we examined the mitochondrial networks of WT and *skn-1b* mutants expressing an outer mitochondrial membrane marker in muscle, *myo-3*::*GFP(mit)*. We found the networks in *skn-1b* mutants to have a disorganised appearance, covering significantly less surface area than that of the WT (Figs 6A–6C and S12C). This is similar to the situation observed in fasted WT animals, implying that *skn-1b* mutants are, at least as far as their mitochondria are concerned, starved (Fig 6A–6C). Fasting *skn-1b* mutants exacerbated these effects on the mitochondrial organisation, indicating that there are also other factors contributing to this mitochondrial morphology phenotype (Fig 6A–6C). A similar pattern was also observed with a second mitochondrial reporter *tomm20*::*GFP* [39] (S12D and S12E Fig). Our data suggest that *skn-1b* contributes to maintaining muscle mitochondrial networks.

We then used transmission electron microscopy to examine mitochondrial morphology more closely. Muscle wall mitochondria from WT and *skn-1b* mutants were compared using sections taken from whole worms. Whist the mitochondria of fed WT animals were rounded, those in the *skn-1b* mutants, were longer and irregular, exhibiting a fused-like state (Figs 6D, 6E and S13). This phenotype could be rescued by re-introducing SKN-1B::GFP into *skn-1b* mutants (Fig 6D). This fused state was also observed in sections from WT fasted animals, supporting the idea that *skn-1b* mutant mitochondria think they are starved (Figs 6D, 6E and S13). However, fasting *skn-1b* mutants caused further deterioration of mitochondrial networks implying that additional factors also contribute to this phenotype (Figs 6D, 6E and S13). In our hands, although the fluorescent images provided evidence of mitochondrial disruption in each case, it was the electron microscopy that showed the precise nature of the disruption (Figs 6A–6E, S14A and S14B). These data together support a model whereby *skn-1b* acts to directly control mitochondrial homeostasis in response to food levels, balancing their fission and fusion.

Mitochondrial membrane proteins are required for mitochondrial fusion and fission: *eat-3*/Opa1 and *fzo-1*/Mfn1 promote fusion and *drp-1*/Drp1 promotes fission [46]. We examined muscle mitochondrial networks, in *C*. *elegans* fed either *eat-3*, *fzo-1* or *drp-1* RNAi using both fluorescent (*myo-3*::*GFP(mit)* and electron microscopy. Mitochondria in animals fed *eat-3* or *fzo-1* RNAi are smaller and more disjointed (as the mitochondria are unable to fuse), whereas those in *drp-1* RNAi fed animals are more elongated (as they cannot fission) (S14A and S14B Fig). Mitochondrial dynamics have previously been implicated in behavioural responses [41]. So, given the behavioural role of *skn-1b* and its importance for maintaining mitochondrial networks, we tested whether the two were linked. Strikingly, we found that whilst neither *eat-3* or *fzo-1* RNAi had any effect on WT exploratory behaviour, both completely rescued *skn-1b* mutant exploration to normal levels (Fig 6F and 6G). *drp-1* RNAi however, had no effect on either WT or *skn-1b* behavioural patterns (Fig 6F and 6G). Together, this supports a model whereby SKN-1B acts to regulate mitochondrial networks, particularly mitochondrial fission, and that this in turn controls food related behaviour.

## Discussion

**Fig 7:**
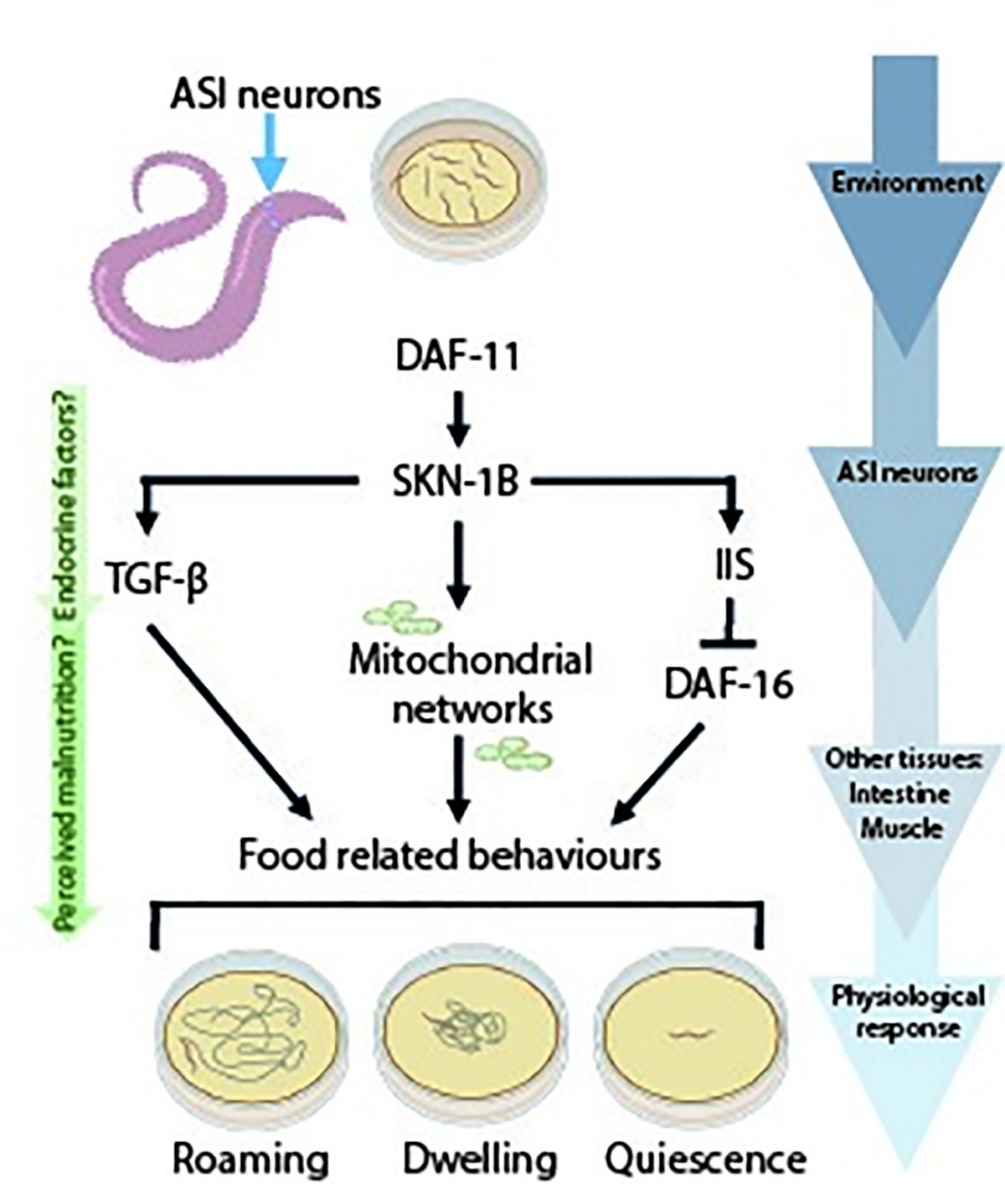
SKN-1B integrates with key nutritional signalling pathways, and regulates mitochondrial networks to control satiety-related behaviour. Food-related behaviour is controlled by interactions between food cues, SKN-1B, downstream signalling pathways (cGMP, TGF-β and IIS), and mitochondria. SKN-1B receives food cues via cGMP signalling (DAF-11). In response to fasting and re-feeding SKN-1B controls satiety quiescence: SKN-1B suppresses *daf-7* expression in the ASIs, downregulating TGF-β signalling and suppressing quiescence (Fig 4A–4C). Fasting also induces DAF-16 nuclear localisation which is maintained after re-feeding to promote quiescence: SKN-1B is required for this response, possibly by acting upstream of both pathways (Fig 5A–5E) [6,7]. In parallel, SKN-1B is also acts to control food-related behaviour by maintaining mitochondrial networks. Overall, this study identifies neuronal SKN-1B as a novel factor in controlling satiety behaviour in response to dietary signals.

Ability to correctly identify a feeling of satiety impacts directly on health. For example, perception of hunger when food is plentiful, can make individuals overeat and gain excess weight, having catastrophic implications for their metabolic status and long-term health [47]. Here we show that in *C*. *elegans*, the transcription factor SKN-1B, regulates satiety behaviour. SKN-1B acts in two hypothalamus-like chemosensory neurons to sense and communicate nutritional status to the rest of the organism. It then controls the animal’s behavioural responses by modulating key nutritional signalling pathways, and maintaining mitochondrial networks (Fig 7).

### Neuronal SKN-1/Nrf mediates the perception of food and satiety

Animals, including *C*. *elegans*, modulate their behaviour by integrating information about their external environment with internal cues. Our data identify SKN-1B as a novel, major regulator of food-related behaviour. SKN-1B levels respond to food availability and memories of fasting events to promote exploration in fed conditions, and suppress quiescence in response to fasting and re-feeding. We propose that SKN-1B acts as a molecular switch, allowing fine-tuning of behaviour in response to environmental change.

It is intriguing that the response of SKN-1B::GFP expression to diet and associated behavioural responses are not always consistent i.e. similar SKN-1B::GFP increases in expression with *B*. *Subtilis* (PY79) and *Pseudomonas* (PA14) leads to opposite behavioural responses (Figs 3A, 3B and S5C). The transcriptional outputs of other SKN-1 isoforms are known to differ depending on the stimulus [35] and it is possible that this is also the case for SKN-1B. Alternatively, in the case of the pathogen response, perhaps additional immune signals are sufficient to override any satiety behaviour in the *skn-1b* mutants.

The constitutively nuclear expression of SKN-1B in ASI neurons (Figs 1D, S3F and S3J) means that it requires the receptor type guanylate cyclase *daf-11* expressed at the amphid opening of the ASI to sense the environment. The expression pattern of *daf-11* and *skn-1b* in the ASI, the requirement of *daf-11* for SKN-1B::GFP expression, and the non-additive behavioural effects of *daf-11* and *skn-1b* strongly imply that these molecules act in the same pathway (Figs 1H, 2A and 3D). *daf-11* has previously been mapped to act upstream of both IIS and TGF-β pathways [5,48,49], and our data identifies a new mode of *daf-11* action (Fig 7). Although *daf-11* and *skn-1b* both act to control quiescence, *daf-11* mutants exhibit decreased quiescence whilst *skn-1b* mutants have increased quiescence compared to WT [5] (Fig 2C and 2D). Therefore, although *daf-11* plays an important role in relation to *skn-1b*’s ties to the environment, it is likely that their behavioural responses to fasting and re-feeding are independent (Fig 7). Complete ablation of the ASIs however actually has the opposite effect to *skn-1b* mutation, reducing satiety-induced quiescence [26]. Thus, genetic removal of SKN-1B does not “break” the neuron. Instead, we propose that specific and rapid changes in SKN-1B levels (Fig 3A–3D) provide sensitivity for modulating behaviour and physiology.

We found that SKN-1B acts specifically to suppress satiety induced quiescence (Fig 2H). In fact, our movement data in fed conditions suggests that *skn-1b* mutants may move slightly more than WT (Fig 2H). As fed *skn-1b* mutants explore less than WT, we could extrapolate that in fed conditions, they spend more time dwelling. Therefore, SKN-1B acts to control different behaviours depending on food status.

### Neuronal SKN-1B modulates TGF-β and IIS to control food-related behaviour

IIS and TGF-β hormone signalling are nutritionally regulated and integral to many processes in worms and mammals. They are regulated by ILPs and NLPs, including the TGF-β ligand DAF-7. In worms they are known to control development, growth, immunity, lifespan and age-related decline [50,51]. Our data suggest that SKN-1B is a sensory switch in the ASIs, acting upstream to modulate both IIS and TGF-β signalling and allowing accurate environmental perception and behavioural control. By regulating DAF-7 in ASIs and DAF-16 in the gut SKN-1B bridges the gap between the external environment and the rest of the worm (Figs 4B, 4C, 5A and 7).

IIS is a conserved pathway for detecting food [52] and reducing IIS using *daf-2* mutants induces quiescence dependent on DAF-16 [53]. Without *skn-1b* however, the contribution of *daf-16* to quiescence is abolished (Fig 5E). Thus, under normal circumstances *skn-1b* allows the worm to achieve appropriate levels of quiescence for its environment (Fig 7). This interaction between *skn-1b* and IIS/*daf-16* was only revealed in the context of rIIS, and under normal conditions the two do not interact genetically to control behaviour (Fig 5B–5E). This suggests to us that in WT *C*. *elegans* ILP signalling originating in the ASIs has to be “programmed” to downregulate IIS for this relationship to be important. Several ILPs could do this, but our data suggest that the insulin receptor agonist INS-7 may be important (S10A and S10B Fig). However, ILPs like INS-7 are differentially expressed in multiple tissues (S10A Fig), and have tissue specific functions making it likely that a complex intercellular network of ILP signalling will be required.

One mechanism via which DAF-16 can regulate quiescence is via food consumption. Worms carrying *daf-2* mutation eat less, and *daf-2; daf-16* double mutants consume more food [54]. Our *skn-1b* mutants have reduced levels of nuclear DAF-16::GFP in their gut, which could simulate a situation comparable to *daf-16* knockdown. However, when fasted and re-fed i.e. conditions that stimulate satiety quiescence, *skn-1b* mutants exhibit higher pharyngeal pumping rates, accumulate more *E*. *coli* in their guts, and are slightly larger than WTs indicating that under these conditions they might be eating more (Fig 2E–2G). In addition, DAF-7 levels are also higher in well fed conditions [26]. Thus, it is possible that altered feeding parameters in *skn-1b* mutants contribute to the increase in *daf-7* reporter expression and satiety induced quiescence behaviour.

### SKN-1B maintains mitochondrial networks to control food-related behaviour

We show SKN-1B acting cell non-autonomously in the gut to alter IIS, and in muscle to alter mitochondrial physiology (Figs 5A, 6A–6E, S12D, S12E, S13 and S14). SKN-1B supports an organised mitochondrial network, balancing fission and fusion to support energy homeostasis in both fed and fasted, re-fed conditions (Figs 6A–6C, S12D and S12E). Mitochondrial homeostasis is implicated in a number of processes including ageing and behaviour. A delicate balance between fission and fusion is necessary for DR to extend lifespan [39]. The fused mitochondria visible in *skn-1b* mutants suggests that SKN-1B acts to control mitochondrial states (Figs 6C–6E and S13). The mitochondrial network observed in *skn-1b* mutants resembles that of fasted or DR worms [39], but it is unlikely that *skn-1b* mutants are physically starved (Fig 2E–2G). We suggest instead, that this occurs via endocrine factors from the ASI leading to a perceived state of malnourishment, with knock-on effects for mitochondrial physiology.

Our data also shows that breaking the fused mitochondrial networks of *skn-1b* mutants using *eat-3* or *fzo-1* RNAi is sufficient to rescue their exploratory behaviour defect (Figs 6F–6H, S14A and S14B). This strongly suggests that SKN-1B mediated control of mitochondrial networks is required for correct behavioural responses to food.

Our work also shows that whilst some DR protocols require *skn-1* to extend lifespan [15], ASI specific *skn-1b* is not essential (Fig 1A and 1B and S1–S3 Tables) [12]. Our results indicate either that the requirement for its expression varies among DR conditions or that there is redundancy for other SKN-1 isoforms in this regard. The role of SKN-1B in regulating mitochondrial networks may also influence the involvement of *skn-1b* in DR longevity [12]. Mitochondrial networks are optimised for ATP production and as such generate increased levels of Reactive Oxygen Species (ROS). Fasting and DR cause mitochondrial fusion and maximise ATP production [8]. Mitochondrial homeostasis is required for DR to extend worm lifespan [39] and, there is also evidence that small increases in ROS increase neuronal SKN-1 expression and promotes longevity [38]. It is possible that *skn-1b* mediated behaviours, influence the impact of DR on lifespan. Different DR protocols cause varying degrees of life extension, and *skn-1b* was required where the increases were modest. As SKN-1B subtly affects feeding this might account for these differences, potentially via changes in *skn-1b* dependent mitochondrial homeostasis.

### Potential for conservation

In mammals, linking food-status to behaviour is controlled by the neuroendocrine system, primarily the hypothalamus: Firstly by the quantity or quality of available food; and secondly by the organism’s internal state i.e. satiety signalling by gut peptides [47]. Our data identifies SKN-1B as a key regulator of satiety quiescence, thought to mimic satiety in mammals [5].

Food levels also alter behaviour in fruit flies, and foraging strategies have been observed that allow adaptation to different food concentrations [55,56]. This suggests that these processes are conserved. Nrfs have been detected in the hypothalamus [57] and some Nrfs also have short isoforms for which functions are not known, suggesting possible conservation. Central Nervous System-specific Nrf1 knockout mice also show neuro-dysfunction phenotypes suggesting that Nrf1 plays an important role here [58]. Our data suggest the interesting possibility that mammalian Nrf proteins might act in the brain to regulate satiety, offering a novel pharmacological target for controlling food-related pathology.

## Methods

### Strains and cloning

Worms were cultured according as previously described [63], and maintained at 20°C unless otherwise indicated. The following strains were used: N2 CGC hermaphrodite stock, GA1058 *skn-1b(tm4241)*, EU1 *skn-1*(zu67), EU31 *skn-1(zu135)*, JMT31 *daf-2(e1370)*, DR1572 *daf-2(e1368)*, DR1574 *daf-2(m1391)*, JMT32 *daf-2(e1370); skn-1b(tm4241)*, GA1060 *daf-2(e1368); skn-1b(tm4241)*, JMT5 *daf-2(e1391); skn-1b(tm4241)*, GA1017 N2 *wuEx217[Pskn-1b*::*skn-1b*::*GFP; rol-6]* (was used for all microscopy and expression analysis), GA1030 *daf-2 wuEx217*, GA1045 *daf-2; daf-16 wuEx217*, GA1034 N2 *wuEx253[Pskn-1b*::*GFP]*, GA1040 *daf-2 wuEx253*, GA1042 *daf-2; daf-16 wuEx253*, DA1116 *eat-2(ad1116)*, JMT7 *eat-2(ad1116); skn-1b(tm4241)*, DR47 *daf-11(m47ts)*, CB1372 *daf-7(e1372)*, JMT68 *daf-7(e1372); skn-1b(tm4241)*, JMT70 *daf-11(m47); skn-1b(tm4241)*, PR678 *tax-4(p678)*, MT1072 *egl-4(n477)*, JMT66 *skn-1b(tm4241) ukcEx15 [Pskn-1b*::*skn-1b*::*GFP; myo-3*::*mcherry]*, JMT67 *ukcEx16 [Pskn-1b*::*skn-1b*::*GFP; myo-3*::*mcherry]*. JMT66 and JMT67 were used for the behavioural rescue experiment as they do not have the roller phenotype. Their expression pattern is identical to that in Fig 1D. COP1836 *knu733[wrmScarlet*::*skn-1b]* (created using CrispR by Knudra Biotech), GA1064 muEx227*[ges-1p*::*GFP*::*daf-16a*], SJ4103 *zIs[myo-3*::*GFP(mit)]*, JMT90 *skn-1b(tm4241) zIs[myo-3*::*GFP(mit)]*, WBM671 *wbmEx289[myo-3p*::*tomm20(aa1-49)*::*GFP*::*unc54 3’UTR]*, JMT76 *skn-1b(tm4241) wbmEx289[myo-3p*::*tomm20(aa1-49)*::*GFP*::*unc54 3’UTR]*, JMT82 *skn-1b(tm4241) muEx227[ges-1p*::*GFP*::*daf-16a];* JMT50 *drcSI7[unc-119;Pdaf-7*::*Venus]*, JMT75 *skn-1b(tm4241) drcSI7[unc-119;Pdaf-7*::*Venus]*. JMT51 *skn-1b(tm4241) wwEx66 [Pins-7*::*GFP + unc- 119(+)]* was made by crossing HT1702 *wwEx66 [Pins-7*::*GFP + unc- 119(+)]* [64] with GA1058 *skn-1b(tm4241)*. Note JMT7 was genotyped used a PCR for *skn-1b(tm4241)* and a pumping rate assay for eat-2. *eat-2* pumping was ~90pumps/min (compared to ~250pumps/min for WT) but no difference in pumping rate (*p* = 0.66) was detected between *eat-2* and *eat-2; skn-1b*. The reporter SKN-1B::GFP reporter was made by cloning a genomic DNA fragment including 2KB directly upstream of the *skn-1b* translational start site, the *skn-1b* genomic region in front of GFP and the endogenous 3’UTR (Fig 1C). It also includes SKN-1D, but as this isoform has not been confirmed *in vivo*, we refer to it as SKN-1B::GFP. However, as intestinal expression of SKN-1B::GFP was not observed in either of our translational reporters under any conditions tested here we conclude that SKN-1B is post translationally modified in the intestine to suppress its expression there. To examine SKN-1C specific expression we also generated a neongreen::SKN-1C CrispR strain (SUNY Biotech). *wuEx217* is used for all SKN-1B::GFP fluorescence microscopy and *ukcEx15* and *ukcEx16* were used for rescue experiments.

### Worm husbandry and lifespan assays

Prior to experiments animals were maintained at the permissive temperature and grown for at least three generations with ample *E*. *coli* food source to assure full viability. Lifespan assays were performed essentially as described [65]. Survival plots and statistical comparisons (Log Rank test) were performed using OASIS2 software [66]. For lifespan assays using RNAi, worms were grown on bacteria expressing the appropriate RNAi clone from the L4 stage. *E*. *coli* HT115 bearing the empty pL4440 vector was used as a control. A summary of the different DR protocols is shown in S3 Table. In some food assays worms were fed different bacterial strains. OP50 and BL21G are *E*. *coli* B strains, HT115,+ W3110 and MG1655 are *E*. *coli* K-12 strains, and HB101 is a B/K-12 hybrid. DA1877 is *Comamonas aquatica* and MyB11 is a *Pseudomonas sp*. encountered in the wild Bacterial isolates from [67–69].

### Microscopy

Fluorescence microscopy: For each slide, 30–40 1 day adult worms were mounted in M9 + 0.06% tetramisole hydrochloride on a 2% agarose pad and imaged within 15 min. Imaging was conducted using a Leica DMR microscope recorded with a Leica-DFC9000GT camera Images are shown at 20x magnification. A 525/50 GFP filter was used and post-processing and quantification was performed using the Fiji distro of ImageJ. For analysing muscle fibres, ImageJ was used to apply a binary threshold to individual muscle fibres and the percentage coverage of GFP-tagged mitochondria across whole fibres calculated as in [39].

Confocal microscopy: day 1 adults were mounted on slides in CyGel (Biostatus) spiked with 0.6% tetramisole hydrochloride to immobilise. Imaging was performed using a Zeiss LSM880/Elyra/Axio Observer.Z1 confocal microscope with the airyscan acquisition mode with the 60x lens. Images were processed with ZenBlue software.

Electron microscopy: 100 L4 worms were picked into M9 buffer. M9 was then aspirated off and replaced by ~2mL 2.5% glutaraldehyde fixative in 100mM sodium cacodylate (CAB) buffer (pH7.2). Worm heads and tails were removed with a scalpel, and the bodies left overnight in fixative at 4°C. Worms were washed twice with CAB and suspended in 2% low melting-point agarose in CAB. Worms were identified in agarose suspension by dissecting scope, excised and transferred to 7mL glass vials, where they were post-fixed in 1% osmium tetroxide in CAB for 1hr at room temperature. These were washed twice in Milli-Q (10 mins each wash), and dehydrated in an ethanol series (50%, 70%, 90% for 10 mins each) followed by 100% dry ethanol (3 times, 10 mins each). Finally samples were washed 2 times (10 mins each) in propylene oxide. Agar scientific low viscosity (LV) resin was prepared fresh and mixed 1:1 with propylene oxide and added to the samples (30 mins RT). Samples were then incubated in fresh LV resin 2 times (2hrs each), embedded in LV resin by polymerising at 60°C for 24hrs. Polymerised samples were identified under a dissecting scope and individual worms were cut out and orientated on a resin block for optimal sectioning. 70nm sections were cut on a Leica EM UC7 ultramicrotome, using a Diatome diamond knife and collected onto 400-mesh copper grids (Agar Scientific). Sections were counterstained with 4.5% uranyl acetate (45 mins) and Reynolds lead citrate (7 mins). Sections were imaged on a Jeol 1230 transmission electron microscope operated at an accelerating voltage of 80kV; images acquired using a Gatan One View 4x4K digital camera.

### Behavioural assays

*C*. *elegans* are genetically tractable, with a characterised nervous system making them an excellent tool to study behaviour. To measure exploration, assays were performed as described [18] (S4A Fig). 35 mm NGM plates were uniformly seeded with 200μL of saturated OP50 culture and allowed to dry overnight at room temperature. Worms were grown in uncrowded conditions to the L4 stage at permissive temperature. Individual L4 animals were placed in the centre of assay plates and transferred to 25°C. After 16 hrs, the animals were tested to see if they were alive by gently touching them, and plates were photographed. Plates were superimposed on a grid of 3.5 mm squares and the number of squares entered by worm tracks counted. Tracks could enter a maximum of 109 squares. At least 15 (one day adult) animals per genotype were tested on three separate days using different offspring generation. Each experiment compared controls and mutants in parallel.

Food/pathogen avoidance assays were performed as described [70]. NGM plates were seeded with 100μL of bacteria culture in the centre of the plate and allowed to dry overnight. Only plates with an evenly and defined circular bacteria lawn were used for the assays. 3 well-developed adult worms from uncrowded plates were transferred to each plate. Animals were allowed to lay eggs for 4hrs at 25°C before being removed from the plates. When animals reached the L4/one day adult stage (48hrs at 25°C) plates were photographed and the numbers of worms on and off the lawn counted (S6A Fig). To measure bordering activity, these images were further analysed to stablish the % of animals on the thicker (outer ~0.5cm) part of the lawn.

Some of these assays required fasting. This was performed as described [5,26] with some modifications. Briefly, animals were maintained either on HB101 or OP50 bacteria at 20°C in non-crowed, non-starved conditions. L4 stage animals were selected and transferred either on HB101 or OP50 seeded plates for 9-12hrs until they have reached young adulthood. Then the animals were transferred with a platinum pick to 60mm NGM plates without food for 16hrs. After 16 hr of fasting, animals were transferred either on HB101 or OP50 bacteria for re-feeding. To measure satiety quiescence animals were fasted for 16hrs and then individuals were transferred to 35mm NGM plates seeded with HB101. Worms were allowed to re-feed for 3 or 6hrs before measuring quiescence. Worms found to be quiescent (cessation of movement/pharyngeal pumping) the duration of this state was measured i.e. until feeding and locomotion resumed. Pumping was measured in individual animals, videoed on food for 1 minute and the pumping rate quantified as in [26].

To assess worm movement with automated technology we worked with Magnitude Biosciences (UK). 60 mm petri dishes containing NGM were seeded with OP50 across the whole area of the dish and a single L4 worm was placed in the centre. Each dish was imaged by 1 of 40 Raspberry Pi Version 2 cameras at a distance of 60 mm from the plate using white transmission illumination from a generic LED light panel. The cameras were located inside a temperature controlled laboratory set to 24°C. For each dish, a sequence of 200 images were taken over a 160secs, with sequences taken every 250secs over 18.3hrs. Each sequence of images was examined for intensity changes corresponding to worm motion, with motion above a threshold level being used to indicate that motion occurred in that burst. The duration of blocks in which movement was not detected were compared with the duration of all blocks recorded to calculate proportion of time in which movement was below the detection threshold and thus the worm was potentially in a quiescent state. A scale factor of 250/160 was applied to extrapolate results to the full length of the experiment. Fiducial markers beyond the plates were imaged to detect global motion caused by external events, and sequences with such global motion were censored from the analysis. Images were processed using version 2.7 of Python programming language and the NumPy library [71].

### Food intake protocol

NGM plates (3.5cm) were seeded with an overnight culture of *E*.*coli* OP50 expressing mCherry. Plates were stored at room temperature for two days. L4 worms were selected and either maintained on OP50 or fasted for 16hrs at 20°C. Fed or fasted worms were then placed on the fluorescent OP50 for 5 minutes and allowed to feed. Worms were imaged and the fluorescence intensity within the gut quantified.

### QPCR

RNA was isolated from adult worms after transfer of the worms to an unseeded NGM plate to remove *E*. *coli*. 50–100 worms were used for each assay. RNA was extracted using Trizol (Sigma) and cDNA synthesized using SuperScript II reverse transcriptase with oligo dT (PCR Biosystems). qRT-PCR was carried out using Fast SYBR Green Master Mix (PCR Biosystems) and the 7900 HT Fast PCR system (PCR Biosystems). Normalization of transcript quantity was carried out using the geometric mean of three stably expressed reference genes Y45F10D.4, *pmp*-3, and *cdc-42* in order to control for cDNA input, as previously described [72]. Primer sequences to detect *skn-1 isoforms*, by qPCR were designed by Primerdesign as follows: *skn-1b* F: aacaggtggatcaacacggc, *skn-1b* R: ttttgcattccaatgtaggc, *skn-1a* F: agtgcttctcttcggtagcc, *skn-1a* R: gaggtgtggacgatggtgaa, *skn-a/c* F: gagagaaggggcacacgacaa, *skn-1a/c* R: tcgagcattctcttcggcag. Statistical analysis was preformed using a student *t*-test.

## Supporting information

S1 Table Data of lifespan trials using DR protocol shown in Fig 1A. Trial 3 is the representative experiment shown in Fig 1A. *eat-2* mutants were long lived in 3 out of 5 trials carried out across 20 and 25°C.
(DOCX)

S2 Table Data of lifespan trials using DR protocol shown in Fig 1B. Trial 1 is the representative experiment shown in Fig 1B.
(DOCX)

S3 Table Summary of the different DR protocols and the involvement of SKN-1. (DOCX)

S4 Table Data of lifespan trials shown in S1 Fig: S1A Fig corresponds to Trial 2 in S1 Table. For S1B–S1F Fig Trial 1 is the representative experiment in each case.
(DOCX)

S5 Table Data of lifespan trials using *daf-2* RNAi shown in S2A and S2B Fig. The experiment was carried out once at each temperature and the data supported the findings of the genetic experiments shown in S1 Table and S1 Fig.
(DOCX)

S1 Fig *skn-1b* is not required for WT or *daf-2* longevity A-F) Survival of WT and *skn-1b* mutants in the absence and presence of *daf-2* mutation. Lower permissive temperatures were used for some *daf-2* alleles as previous work showed that *skn-1c* had a stronger suppressive effect on *daf-2* at these compared to higher temperatures [10, 16]. Representative experiments shown, individual trials are summarised with Log-Rank analysis in S4 Table. NB: In a total of 13 lifespan trials, we observed that *skn-1b* mutation partially suppressed *daf-2* longevity in only 4 trials (S4 Table). An additional two trials using *daf-2* RNAi did not require *skn-1b* (S2A and S2B Fig and S5 Table). We conclude that *skn-1b* does not contribute to *daf-2* longevity. In a total of 15 trials (13 on OP50, 2 on HT115) bacteria we observed a slight decrease in *skn-1b* longevity compared to WT in 4 trials (S4 and S5 Tables). We conclude that *skn-1b* does not contribute to normal lifespan. *skn-1b(tm4241)* allele details (S5 Fig).
(TIF)

S2 Fig *skn-1b* is not required for *daf-2* RNAi incurred longevity. **A-B)** Survival of WT and *skn-1b* mutants in the absence and presence of *daf-2* RNAi. Full data for each trial are summarised together with Log-Rank analysis in S5 Table.
(TIF)

S3 Fig Characterisation of *skn-1b(tm4241)* and SKN-1B:GFP. **A)** Expression levels of *skn-1* isoforms in WT and *skn-1b(tm4241)* determined by Q-PCR. Combined data from 6 biological replicates shown. Error bars show st. dev. Two-tailed t-test compared to WT control *p<0.05, **p< 0.001, ***p<0.0001, NS not significant. **B-D)** Brood size of WT and *skn-1b* mutants at three different temperatures. *skn-1b* mutants are fully fertile, so can be maintained as homozygotes. Combined data from 3 biological replicates shown, n>30 worms per group. Error bars show st. dev. Two-tailed t-test *p<0.05, **p< 0.001, ***p<0.0001, NS not significant. **E)** Age-specific fecundity in WT and *skn-1b* mutants. **F)** Expression pattern of the Scarlet::SKN-1B reporter in day 1 adults under fed conditions shows SKN-1B in ASI neurons. Our lab also generated an endogenous NeonGreen::SKN-1C reporter but cannot detect expression of SKN-1C in neurons (available on request). No significant differences were observed using a two-tailed t-test on any day between genotypes. **G-J)** SKN-1B::GFP is expressed differentially during growth and in additional neurons in response to bacterial deprivation. H) SKN-1B::GFP observed in additional neurons in response to fasting. **I)** Quantification of the number of visible neurons in SKN-1B::GFP expressing worms in response to fasting. A total of 52 fed and 64 fasted worms were examined. **J)** DiI staining confirms SKN-1B::GFP in the ASI neurons and identified two of these additional neurons (counted in S3I Fig) as being the ADLs. Recently, others have identified SKN-1B in AIY neurons as a regulator of chemosensory processes and behaviour, showing that animals lacking the *skn-1a*, *c* and *b* do not chemotax towards NaCl, butanone or temperature, or move towards thicker bacterial lawns (as a WT worms would) [57]. We tested our *skn-1b* specific mutant in a NaCl chemotaxis assay and got similar results but have no evidence that SKN-1B is endogenously expressed in the AIYs in fed conditions. It is possible that SKN-1B signals from the ASI—AIY neurons to mediate this response or that some SKN-1B::GFP expressing neurons in fasted conditions are AIYs. As the no-food conditions in NaCl chemotaxis assays is sufficient to induce SKN-1B expression in these cells, this could mediate the effects.
(TIF)

S4 Fig Role of *skn-1b* in regulating exploratory behaviour. **A)** Cartoon showing setup of exploration assay as in [18]. Extended dwelling or roaming compared to WT behaviour can be quantified by counting the number of squares that a worm traverses over 16hrs. Food is spread evenly and continuously on the plate. **B)** Control experiments for exploration assay. Time spent in roaming and dwelling states depends on integrating internal neuro-modulatory cues with external sensory cues. The absence of such sensory transduction leads to extended dwelling as observed in the *tax-4* mutant. *tax-4* encodes a cyclic nucleotide-gated channel subunit, in contrast, mutants with constitutive sensory input, such as the *egl-4*, which encodes a molecule with cGMP protein kinase activity, exhibit extended roaming [73]. Representative experiment of 3 biological replicates shown, n<15 worms per group ± st. dev. **C and D)** Quantification of exploratory behaviour in response to *skn-1* RNAi fed at either the L1 **(C)** or L4 stage **(D)**. Mean plate coverage of n>23 individual worms per group ± st. dev., one representative experiment of 3 biological replicates shown. For **B-D)** Two-tailed t-test NS non-significant. Two-tailed t-test *p<0.05, **p<0.001, ***p<0.0001, NS not significant. **E)** Neurons are relatively resistant to RNAi (Timmons et al., 2001), but quantitative fluorescence microscopy shows that *skn-1* RNAi from the L1 stage reduces SKN-1B::GFP in ASIs by ~30%. The smaller difference between exploration in WT and *skn-1b* mutants in **(D)** likely reflects the RNAi knock-down from L4 being less complete. The *skn-1* RNAi clone used in **C-E** targets all *skn-1* isoforms [16]. Pooled data from 3 biological replicates shown, n>100 individual worms per group. Two-tailed t-test ***p<0.0001. **F)** *skn-1b* mutants display normal thrashing activity in liquid. Average of 3 biological replicates shown, n>33 individual worms per group.
(TIF)

S5 Fig Effect of *skn-1b* mutation on food avoidance behaviour. **A)** Cartoon showing setup of food avoidance assay as in [70]. The percentage of worms on a lawn of bacteria is determined in conditions where worms have a choice whether to be on or off the lawn. **B-C)** Quantification of worms on different bacterial lawns (if given a choice to leave). Other strains tested are shown in Fig 1I. Each bar represents a mean of 3 biological replicates with ~100 worms per trial ± st. dev. Two-tailed t-test *p<0.05, **p< 0.001, ***p<0.0001, NS not significant. For **B-C)** bacteria were allowed to proliferate in each case, and no antibiotics or FUDR were present (see Methods). NB: Our assay measures satiety quiescence following fasting, as this offers an easily quantifiable behaviour. However, satiety quiescence also occurs cyclically between foraging and dwelling when worms are fully fed. During our studies we observed that while *skn-1b* mutants always preferred the bacterial lawn regardless of the food type, WT worms could be tempted to spend more time on certain bacteria (Fig 1I). A similar defect in food sensing behaviour was also observed for *skn-1* mutants [57].
(TIF)

S6 Fig Role of *skn-1b* in mediating entry to quiescence. **A-F)** % worms spending time in quiescence 3 or 6hrs after fasting and re-feeding. Each bar represents a mean of 3 biological replicates ± SEM with n>36 worms per group. Due to the nature of the assay, satiety quiescence is not observable in every worm in an experiment, particularly in mutant strains that exhibit low levels of quiescence such as *daf-7* and *daf-11*. Similar numbers of worms from WT and mutants were observed in quiescence (S6C Fig) but fewer *daf-7* and *daf-7;skn-1b* mutants entered quiescence (S6C Fig). Thus, the *daf-7* data in Fig 4A is likely to be an over-representation of the actual level of satiety quiescence within the population.
(TIF)

S7 Fig SKN-1B expression and response to environmental change. **A-C)** Quantitative fluorescence microscopy of SKN-1B::GFP expression in response to pathogenic bacteria **(A)**, an alternative DR protocol (Moroz et al 2014) **(B)**, or *eat-2* mutation **(C)**. For **(A and C)** bacteria were allowed to proliferate in each case, in **B)** antibiotics were present (see Methods). For **A-C)** Error bars show st. dev. Two-tailed t-test compared to day 1 expression levels *p<0.05, **p< 0.001, ***p<0.0001, NS not significant.
(TIF)

S8 Fig Epistasis of *daf-7* and *skn-1b* in regulating exploration. **A-E)** Individual exploration assays combined in Fig 4D. We reasoned that if each of the two genes regulate different behaviours independently, then the effects of *daf-7* and *skn-1b* on behaviour should be additive. However, the exploration of *daf-7* and *daf-7; skn-1b* worms was not significantly changed in 4 out of 5 trials i.e. not additive effect. In each experiment the mean plate coverage of n>8 individual worms per group is shown ± standard deviation. Two-tailed t-test *p<0.05, **p< 0.001, ***p<0.0001, NS not significant. **F)** Statistical analysis of each individual and combined trial(s). Comparisons highlighted in green are significant (two-tailed t-test p<0.05), and those in orange are NS.
(TIF)

S9 Fig *skn-1b* alters DAF-16::GFP nuclear localisation in response to diet changes. **A)** Scoring system for DAF-16a::GFP nuclear localisation in the gut nuclei. Nuclear localisation was graded by a four-point system; 0 = none, 1 = low, 2 = intermediate, 3 = high. Nuclear grading was carried out by a combination of the quantity of punctate gut nuclei as well as the fluorescence intensity of these nuclei. **B)** Quantification of the grading of the DAF-16a::GFP nuclear localisation in both WT and *skn-1b* mutants under fed, fasted, fasted/re-fed for 3hrs or 6hrs. **C)** Full statistical analysis using two tailed t-test for the average grading of DAF-16a::GFP nuclear localisation shown in Fig 5A. **D)** Full statistical analysis determined by chi-squared test of DAF-16 nuclear localisation data shown in S9B Fig.
(TIF)

S10 Fig *Pins-7*::*GFP* levels are altered in *skn-1b* mutants. **A)** Representative images showing *Pins-7*::*GFP* expression in WT and *skn-1b* mutants. Expression was visible in various neurons and the gut. 20x magnification. **B)** Quantitative fluorescence microscopy of *Pins-7*::*GFP* in the gut. Neuronal *Pins-7*::*GFP* levels were not quantified as its expression in multiple neurons made their individual identification difficult. *skn-1* has also been shown to repress *ins-7* expression [35], consistent with the increase in *ins-7*::*GFP* observed in *skn-1b* animals.
(TIF)

S11 Fig Interaction of *skn-1b* and IIS for exploratory behaviour. **A and B)** Quantification of exploration. One representative experiment of 3 similar biological replicates shown ± st. dev., n>10 worms per group. Two-tailed t-test *p<0.05, **p< 0.001, ***p<0.0001, NS not significant. *daf-2(e1368)* caused a milder exploratory defect than *daf-2(e1370)* (Fig 5B). Fasting also reduced exploration in *daf-2(e1368)* animals but this was not further reduced by *skn-1b* mutation (Fig 5C). Therefore, despite this milder *daf-2(e1368)* exploratory phenotype, *skn-1b* mutation was not able to further suppress exploration in either fed, or fasted and re-fed conditions.
(TIF)

S12 Fig Comparing mitochondria in WT and *skn-1b* mutants—supporting data. **A and B)** Images and quantification of bordering behaviour. Each bar represents a mean of 3 biological replicates ± st. dev. **C)** Scoring system of the expression of myo-3::mitoGFP in *C*. *elegans*. **D and E)** Expression and quantification of WT and *skn-1b* mutant *C*. *elegans* expressing tomm20:GFP. This reporter expresses a peptide of tomm20, an outer mitochondrial membrane protein and hence marks all mitochondria, delineating their shape [39]. In **E)** Each bar represents a mean of 3 biological replicates ± SEM, n>49 day 1 adults worms per group. Note that although fluorescence microscopy demonstrates an alteration in the organisation of the mitochondrial networks and suggests a level of disruption, it is comparison with TEM (Figs 6D, S12, S13 and S14) that allowed us to define the nature of the disruption.
(TIF)

S13 Fig TEM of WT and *skn-1b* mutants in Fed and fasted conditions. **A)** Longitudinal sections and **B)** Transverse sections. All scale bars = 500nm, M = mitochondria, S = sarcomere. Fasting disrupts mitochondrial networks in response to fasting in WT animals. *skn-1b* mutants also have disrupted mitochondrial networks, exhibiting increased fusion of mitochondria. In response to fasting *skn-1b* mutant mitochondria appear much worse than WT, with disrupted membranes and cristae structures.
(TIF)

S14 Fig Comparing mitochondrial networks in WT and *skn-1b* mutants using fluorescent and Electron microscopy. Images of myo-3::mitoGFP **(A)** and TEM images **(B)** in WT and *skn-1b* mutant *C*. *elegans* fed control, *eat-3*, *fzo-1* or *drp-1* RNAi. TEM shows Longitudinal sections 200nm. Note that although the fluorescent images in A clearly show signs of mitochondrial network disruption, it is only when examining the TEM images that the precise network structures can be seen e.g. both *eat-3* and *drp-1* RNAi show a “spotty” pattern on the fluorescent images but this translates to a very different TEM image with *eat-3* RNAi causing fission and *drp-1* RNAi fusion (as expected).
(TIF)
