# Supplementary material for: Neuronal SKN-1B modulates nutritional signalling pathways and mitochondrial networks to control satiety
Source: PLoS Genet. 2021 Mar 4;17(3):e1009358. doi: 10.1371/journal.pgen.1009358 (PMC7932105; doi:10.1371/journal.pgen.1009358)
Supplement: S1 Table — Trial 3 is the representative experiment shown in Fig 1A. eat-2 mutants were long lived in 3 out of 5 trials carried out across 20 and 25°C. (DOCX) [file pgen.1009358.s001.docx]

# **S1 Table**

| **Trial** | **Strain** | **Genotype** | **Mean Lifespan (days)** | **Temp**  **°C** | **Extension (%)** | **p value**  **(Log-rank)**  **vs** | **n dead (total)** |
| --- | --- | --- | --- | --- | --- | --- | --- |
| 1 | WT |  | 12.47 | 25 |  |  | 60 |
| 1 | GA1058 | *skn-1b(tm4241)* | 13.05 | 25 | +4.6 | WT:NS | 82 |
| 1 | DA1116 | *eat-2(ad1116)* | 13.82 | 25 | +10.8 | WT: <0.05 | 84 |
| 1 | JMT7 | *eat-2; skn-1b* | 12.18 | 25 | -2.3 | WT:NS  DA1116:<0.0001 | 65 |
|  |  |  |  |  |  |  |  |
| 2 | WT |  | 11.42 | 25 |  |  | 55 |
| 2 | GA1058 | *skn-1b(tm4241)* | 12.07 | 25 | +5.7 | WT:NS | 67 |
| 2 | DA1116 | *eat-2(ad1116)* | 11.99 | 25 | +4.9 | WT:NS | 81 |
| 2 | JMT7 | *eat-2; skn-1b* | 11.67 | 25 | +2.2 | WT:NS  DA1116:NS | 73 |
|  |  |  |  |  |  |  |  |
| 3 | WT |  | 11.04 | 25 |  |  | 84 |
| 3 | GA1058 | *skn-1b(tm4241)* | 11.6 | 25 | +5.1 | WT:NS | 75 |
| 3 | DA1116 | *eat-2(ad1116)* | 12.67 | 25 | +14.8 | WT:<0.0001 | 81 |
| 3 | JMT7 | *eat-2; skn-1b* | 11.14 | 25 | +0.9 | WT:NS  DA1116: <0.0001 | 74 |
|  |  |  |  |  |  |  |  |
| 4 | WT |  | 17.72 | 20 |  |  | 91 |
| 4 | GA1058 | *skn-1b(tm4241)* | 19 | 20 | +7.22 | WT:NS | 41 |
| 4 | DA1116 | *eat-2(ad1116)* | 24.68 | 20 | +39.3 | WT:<0.0001 | 50 |
| 4 | JMT7 | *eat-2; skn-1b* | 22.51 | 20 | +27 | WT:<0.0001  DA1116:<0.05 | 74 |
|  |  |  |  |  |  |  |  |
| 5 | WT |  | 28.42 | 20 |  |  | 60 |
| 5 | GA1058 | *skn-1b(tm4241)* | 24.14 | 20 | -12 | WT<0.05 | 48 |
| 5 | DA1116 | *eat-2(ad1116)* | 23.57 | 20 | -17.1 | WT:<0.0001 | 58 |
| 5 | JMT7 | *eat-2; skn-1b* | 24.88 | 20 | -12.5 | WT:<0.05  DA1116:NS | 49 |
