# Supplementary material for: Neuronal SKN-1B modulates nutritional signalling pathways and mitochondrial networks to control satiety
Source: PLoS Genet. 2021 Mar 4;17(3):e1009358. doi: 10.1371/journal.pgen.1009358 (PMC7932105; doi:10.1371/journal.pgen.1009358)
Supplement: S2 Table — Trial 1 is the representative experiment shown in Fig 1B. (DOCX) [file pgen.1009358.s002.docx]

# **S2 Table**

| **Trial** | **Strain** | **Genotype** | **Mean Lifespan (days)** | **Temp**  **°C** | **Extension (%)** | **p value**  **(Log-rank)**  **vs** | **n dead (total)** |
| --- | --- | --- | --- | --- | --- | --- | --- |
| 1 AL | WT |  | 28.43 | 20 |  |  | 136 |
| 1 DR | WT |  | 43.74 | 20 | +53.8 | WT AL:<0.0001 | 150 |
| 1 AL | GA1058 | *skn-1b(tm4241)* | 28.58 | 20 | +0.5 | WT AL:NS | 118 |
| 1 DR | GA1058 | *skn-1b(tm4241)* | 42.79 | 20 | +50.5 | WT AL:<0.0001  WT DR: NS  GA1058 AL:<0.0001 | 116 |
| 1 AL | WT |  | 25.81 | 20 |  |  | 102 |
| 1 DR | WT |  | 48.53 | 20 | +88.0 | WT AL:<0.0001 | 93 |
| 1 AL | GA1058 | *skn-1b(tm4241)* | 26.17 | 20 | +1.4 | WT AL:NS | 104 |
| 1 DR | GA1058 | *skn-1b(tm4241)* | 41.83 | 20 | +62.1 | WT AL:<0.0001  WT DR: <0.0001  GA1058 AL:<0.0001 | 114 |
