# Supplementary material for: Neuronal SKN-1B modulates nutritional signalling pathways and mitochondrial networks to control satiety
Source: PLoS Genet. 2021 Mar 4;17(3):e1009358. doi: 10.1371/journal.pgen.1009358 (PMC7932105; doi:10.1371/journal.pgen.1009358)
Supplement: S3 Table — (DOCX) [file pgen.1009358.s003.docx]

# **S3 Table**

**References**

1. Moroz N, Carmona JJ, Anderson E, Hart AC, Sinclair D a., Blackwell TK. Dietary restriction involves NAD+ -dependent mechanisms and a shift toward oxidative metabolism. Aging Cell. 2014;13: 1075–1085. doi:10.1111/acel.12273

2. Bishop N a, Guarente L. Two neurons mediate diet-restriction-induced longevity in C. elegans. Nature. 2007;447: 545–549. doi:10.1038/nature05904

3. Tang L, Choe KP. Characterization of skn-1/wdr-23 phenotypes in Caenorhabditis elegans; pleitrophy, aging, glutathione, and interactions with other longevity pathways. Mech Ageing Dev. 2015;149: 88–98. doi:10.1016/j.mad.2015.06.001

| **Description and % extension** | **Bacteria** | **Additions** | **Reported SKN-1 requirement** |
| --- | --- | --- | --- |
| Liquid  DR from day 3 adults  ~60% extension | OP50 | ﻿Amp, Kan, Tet, Nystatin, FUdR | Yes  No LS Extension in *skn-1 (zu135)*  [1] |
| Liquid on top of solid agar  DR from L4/day 1 adults  ~22% extension | HT115 | Erythromycin, Amp, FUdR, IPTG | Yes  *skn-1(zu135)* cannot respond to DR  Rescued with SKN-1B in ASI  [1,2] |
| Genetic (continuous) DR  *eat-2(ad1116)*  Solid media  20-60% extension | OP50 | Nystatin, FUdR | Yes, partial  *skn-1(zu67)* can only partially respond to DR  [3] |
