# Supplementary material for: Neuronal SKN-1B modulates nutritional signalling pathways and mitochondrial networks to control satiety
Source: PLoS Genet. 2021 Mar 4;17(3):e1009358. doi: 10.1371/journal.pgen.1009358 (PMC7932105; doi:10.1371/journal.pgen.1009358)
Supplement: S4 Table — For S1B–S1F Fig Trial 1 is the representative experiment in each case. (DOCX) [file pgen.1009358.s004.docx]

# **S4 Table**

| **Trial** | **Strain** | **Genotype** | **Mean Lifespan (days)** | **Temp**  **°C** | **Extension (%)** | ***P* value**  **(Log rank)**  **vs** | **n dead**  **(total)** |
| --- | --- | --- | --- | --- | --- | --- | --- |
| 1 | N2 |  | 30.42 | 15 |  |  | 79 (115) |
| 1 | JMT31 | *daf-2(e1370)* | 45.94 | 15 | +51.0 | N2: <0.0001  JMT32: <0.0001 | 33 (48) |
| 1 | JMT32 | *daf-2; skn-1b* | 35.81 | 15 | +35.81 | N2: <0.05  JMT31: <0.0001 | 55 (75) |
|  |  |  |  |  |  |  |  |
| 2 | N2 |  | 30.67 | 15 |  |  | 31 (43) |
| 2 | GA1058 | *skn-1b(tm4241)* | 27.63 | 15 | -9.9 | N2: NS  JMT31: <0.0001  JMT32: <0.0001 | 51 (82) |
| 2 | JMT31 | *daf-2(e1370)* | 40.86 | 15 | +33.2 | N2: <0.0001  GA1058: <0.0001  JMT32: NS | 49 (64) |
| 2 | JMT32 | *daf-2; skn-1b* | 43.42 | 15 | +41.2 | N2: <0.0001  GA1058: <0.0001  JMT31: NS | 57 (85) |
|  |  |  |  |  |  |  |  |
| 3 | N2 |  | 31.49 | 15 |  |  | 61 (73) |
| 3 | GA1058 | *skn-1b(tm4241)* | 27.49 | 15 | -12.8 | N2: NS  JMT31: <0.0001  JMT32: <0.0001 | 33 (44) |
| 3 | JMT31 | *daf-2(e1370)* | 44.50 | 15 | +41.3 | N2: <0.0001  GA1058: <0.0001  JMT32: NS | 24 (38) |
| 3 | JMT32 | *daf-2; skn-1b* | 39.32 | 15 | +24.9 | N2: <0.01  GA1058: <0.0001  JMT31: NS | 54 (79) |
|  |  |  |  |  |  |  |  |
| 1 | N2 |  | 22.27 | 20 |  |  | 77 (105) |
| 1 | GA1058 | *skn-1b(tm4241)* | 22.64 | 20 | +1.7 | N2: NS  JMT31: <0.0001  JMT32: <0.0001 | 78 (93) |
| 1 | JMT31 | *daf-2(e1370)* | 40.25 | 20 | +80.7 | N2: <0.0001  GA1058: <0.0001  JMT32: NS | 71 (88) |
| 1 | JMT32 | *daf-2; skn-1b* | 36.97 | 20 | +66 | N2: <0.0001  GA1058: <0.0001  JMT31: NS | 104 (116) |
|  |  |  |  |  |  |  |  |
| 2 | N2 |  | 22.25 | 20 |  |  | 58 (69) |
| 2 | GA1058 | *skn-1b(tm4241)* | 19.86 | 20 | -1.7 | N2: NS  JMT31: <0.0001  JMT32: <0.0001 | 43 (53) |
| 2 | JMT31 | *daf-2(e1370)* | 39.26 | 20 | +76.4 | N2: <0.0001  GA1058: <0.0001  JMT32: NS | 74 (85) |
| 2 | JMT32 | *daf-2; skn-1b* | 36.48 | 20 | +64 | N2: <0.0001  GA1058: <0.0001  JMT31: NS | 62 (69) |
|  |  |  |  |  |  |  |  |
| 1 | N2 |  | 20.66 | 20 |  |  | 44 (50) |
| 1 | GA1058 | *skn-1b(tm4241)* | 19.95 | 20 | -3.43659 | N2: NS  DR1572: <0.0001  GA1060: <0.0001 | 53 (72) |
| 1 | DR1572 | *daf-2(e1368)* | 30.45 | 20 | +47.4 | N2: <0.0001  GA1058: <0.0001  GA1060: NS | 45 (92) |
| 1 | GA1060 | *daf-2; skn-1b* | 29.57 | 20 | +43.1 | N2: <0.0001  GA1058: <0.0001  DR1572: NS | 57 (73) |
|  |  |  |  |  |  |  |  |
| 2 | N2 |  | 25.29 | 20 |  |  | 88 (125) |
| 2 | GA1058 | *skn-1b(tm4241)* | 22.46 | 20 | -11.2 | N2: <0.05  DR1572: <0.0001  GA1060: <0.0001 | 78 (112) |
| 2 | DR1572 | *daf-2(e1368)* | 34.04 | 20 | +34.6 | N2: <0.0001  GA1058: <0.0001  GA1060: NS | 68 (94) |
| 2 | GA1060 | *daf-2; skn-1b* | 32.47 | 20 | +28.4 | N2: <0.0001  GA1058: <0.0001  DR1572: NS | 38 (59) |
|  |  |  |  |  |  |  |  |
| 1 | N2 |  | 12.99 | 25 |  |  | 68 (79) |
| 1 | GA1058 | *skn-1b(tm4241)* | 13.26 | 25 | +2.1 | N2: NS  DR1572: <0.0001  GA1060: <0.0001 | 96 (100) |
| 1 | DR1572 | *daf-2(e1368)* | 21.32 | 25 | +64.1 | N2: <0.0001  GA1058: <0.0001  GA1060: <0.05 | 68 (94) |
| 1 | GA1060 | *daf-2; skn-1b* | 20.02 | 25 | +54.1 | N2: <0.0001  GA1058: <0.0001  DR1572: <0.05 | 72 (98) |
|  |  |  |  |  |  |  |  |
| 2 | N2 |  | 13.07 | 25 |  |  | 81 (88) |
| 2 | GA1058 | *skn-1b(tm4241)* | 13.63 | 25 | +4.3 | N2: NS  DR1572: <0.0001  GA1060: <0.0001 | 107 (119) |
| 2 | DR1572 | *daf-2(e1368)* | 17.47 | 25 | +33.7 | N2: <0.0001  GA1058: <0.0001  GA1060: NS | 36 (38) |
| 2 | GA1060 | *daf-2; skn-1b* | 16.31 | 25 | +24.8 | N2: <0.0001  GA1058: <0.0001  DR1572: NS | 52 (57) |
|  |  |  |  |  |  |  |  |
| 1 | N2 |  | 28.87 | 15 |  |  | 120(127) |
| 1 | GA1058 | *skn-1b(tm4241)* | 24.99 | 15 | -13.4 | N2: <0.05  DR1574: <0.0001  JMT5: <0.0001 | 80 (98) |
| 1 | DR1574 | *daf-2(e1391)* | 40.54 | 15 | +40.4 | N2: <0.0001  GA1058: <0.0001  JMT5: NS | 45(57) |
| 1 | JMT5 | *daf-2; skn-1b* | 36.57 | 15 | +26.67 | N2: <0.0001  GA1058: <0.0001  DR1574: NS | 82(100) |
|  |  |  |  |  |  |  |  |
| 2 | N2 |  | 28.05 | 15 |  |  | 79 (105) |
| 2 | GA1058 | *skn-1b(tm4241)* | 26.51 | 15 | -5.5 | N2: NS  DR1574: <0.0001  JMT5: <0.0001 | 66 (87) |
| 2 | DR1574 | *daf-2(e1391)* | 41.55 | 15 | +48.1 | N2: <0.0001  GA1058: <0.0001  JMT5: <0.05 | 99 (123) |
| 2 | JMT5 | *daf-2; skn-1b* | 49.04 | 15 | +74.8 | N2: <0.0001  GA1058: <0.0001  DR1574: <0.05 | 28 (38) |
|  |  |  |  |  |  |  |  |
| 1 | N2 |  | 23.05 | 20 |  |  | 79 (92) |
| 1 | GA1058 | *skn-1b(tm4241)* | 23.46 | 20 | +1.8 | N2: NS  DR1574: <0.0001  JMT5: <0.0001 | 89 (96) |
| 1 | DR1574 | *daf-2(e1391)* | 44.35 | 20 | +92.4 | N2: <0.0001  GA1058: <0.0001  JMT5: NS | 77 (102) |
| 1 | JMT5 | *daf-2; skn-1b* | 39.12 | 20 | +69.7 | N2: <0.0001  GA1058: <0.0001  DR1574: NS | 58 (78) |
|  |  |  |  |  |  |  |  |
| 2 | N2 |  | 23.77 | 20 |  |  | 89 (93) |
| 2 | GA1058 | *skn-1b(tm4241)* | 19.8 | 20 | -16.7 | N2: <0.0001  DR1574: <0.0001  JMT5: <0.0001 | 53 (57) |
| 2 | DR1574 | *daf-2(e1391)* | 39.73 | 20 | +67.1 | N2: <0.0001  GA1058: <0.0001  JMT5: <0.05 | 89 (139) |
| 2 | JMT5 | *daf-2; skn-1b* | 33.54 | 20 | +41.1 | N2: <0.0001  GA1058: <0.0001  DR1574: <0.05 | 60 (82) |
