# Supplementary material for: Neuronal SKN-1B modulates nutritional signalling pathways and mitochondrial networks to control satiety
Source: PLoS Genet. 2021 Mar 4;17(3):e1009358. doi: 10.1371/journal.pgen.1009358 (PMC7932105; doi:10.1371/journal.pgen.1009358)
Supplement: S5 Table — The experiment was carried out once at each temperature and the data supported the findings of the genetic experiments shown in S1 Table and S1 Fig. (DOCX) [file pgen.1009358.s005.docx]

# **S5 Table**

| **Trial** | **Strain** | **Genotype** | **Mean Lifespan (days)** | **Temp**  **°C** | **Extension (%)** | ***P* value**  **(Log rank)**  **vs** | **n dead**  **(total)** |
| --- | --- | --- | --- | --- | --- | --- | --- |
| 1 | N2 | Control RNAi | 28.56 | 15 |  |  | 99 (100) |
| 1 | N2 | *daf-2* RNAi | 47.74 | 15 | +59.8 | ^N2 C:<0.001^ | 93 (100) |
| 1 | GA1058 | Control RNAi | 23.84 | 15 | -19.7 | ^N2 C:<0.01^ | 100 (100) |
| 1 | GA1058 | *daf-2* RNAi | 47.93 | 15 | +59.3 | ^N2 C: <0.001^  ^N2 d: NS^  ^GA1058 C: <0.001^ | 93 (100) |
|  |  |  |  |  |  |  |  |
| 1 | N2 | Control RNAi | 20.66 | 20 |  |  | 100 (100) |
| 1 | N2 | *daf-2* RNAi | 35.05 | 20 | +58.9 | ^N2 C: NS^ | 97 (100) |
| 1 | GA1058 | Control RNAi | 21.54 | 20 | +4.2 | ^N2 C: <0.001^ | 100 (100) |
| 1 | GA1058 | *daf-2* RNAi | 35.63 | 20 | +57.9 | ^N2 C: <0.001^  ^N2 d: NS^  ^GA1058 C: <0.001^ | 97 (100) |
