# Supplementary figures and images for: Neuronal SKN-1B modulates nutritional signalling pathways and mitochondrial networks to control satiety
Source: PLoS Genet. 2021 Mar 4;17(3):e1009358. doi: 10.1371/journal.pgen.1009358 (PMC7932105; doi:10.1371/journal.pgen.1009358)

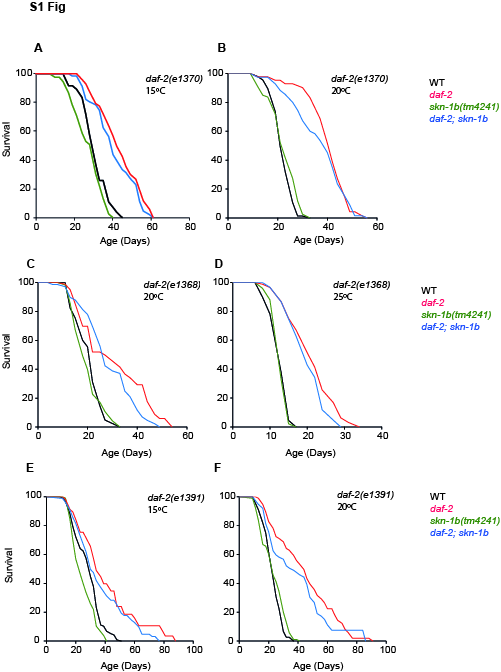

Supplement: S1 Fig — Lower permissive temperatures were used for some daf-2 alleles as previous work showed that skn-1c had a stronger suppressive effect on daf-2 at these compared to higher temperatures [10, 16]. Representative experiments shown, individual trials are summarised with Log-Rank analysis in S4 Table. NB: In a total of 13 lifespan trials, we observed that skn-1b mutation partially suppressed daf-2 longevity in only 4 trials (S4 Table). An additional two trials using daf-2 RNAi did not require skn-1b (S2A and S2B Fig and S5 Table). We conclude that skn-1b does not contribute to daf-2 longevity. In a total of 15 trials (13 on OP50, 2 on HT115) bacteria we observed a slight decrease in skn-1b longevity compared to WT in 4 trials (S4 and S5 Tables). We conclude that skn-1b does not contribute to normal lifespan. skn-1b(tm4241) allele details (S5 Fig). (TIF) [file pgen.1009358.s006.tif]

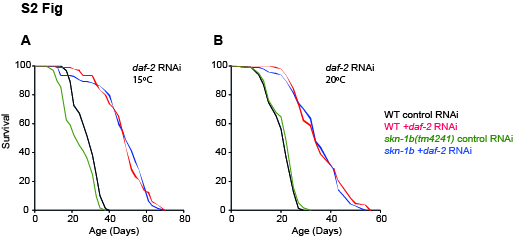

Supplement: S2 Fig — A-B) Survival of WT and skn-1b mutants in the absence and presence of daf-2 RNAi. Full data for each trial are summarised together with Log-Rank analysis in S5 Table. (TIF) [file pgen.1009358.s007.tif]

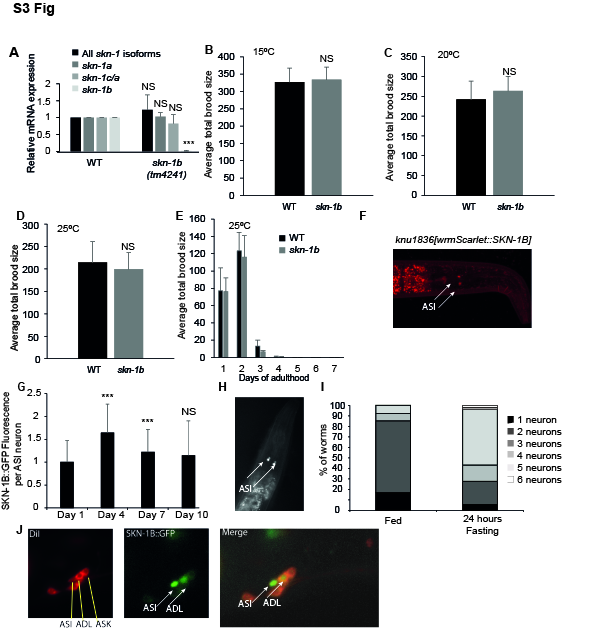

Supplement: S3 Fig — A) Expression levels of skn-1 isoforms in WT and skn-1b(tm4241) determined by Q-PCR. Combined data from 6 biological replicates shown. Error bars show st. dev. Two-tailed t-test compared to WT control *p<0.05, **p< 0.001, ***p<0.0001, NS not significant. B-D) Brood size of WT and skn-1b mutants at three different temperatures. skn-1b mutants are fully fertile, so can be maintained as homozygotes. Combined data from 3 biological replicates shown, n>30 worms per group. Error bars show st. dev. Two-tailed t-test *p<0.05, **p< 0.001, ***p<0.0001, NS not significant. E) Age-specific fecundity in WT and skn-1b mutants. F) Expression pattern of the Scarlet::SKN-1B reporter in day 1 adults under fed conditions shows SKN-1B in ASI neurons. Our lab also generated an endogenous NeonGreen::SKN-1C reporter but cannot detect expression of SKN-1C in neurons (available on request). No significant differences were observed using a two-tailed t-test on any day between genotypes. G-J) SKN-1B::GFP is expressed differentially during growth and in additional neurons in response to bacterial deprivation. H) SKN-1B::GFP observed in additional neurons in response to fasting. I) Quantification of the number of visible neurons in SKN-1B::GFP expressing worms in response to fasting. A total of 52 fed and 64 fasted worms were examined. J) DiI staining confirms SKN-1B::GFP in the ASI neurons and identified two of these additional neurons (counted in S3I Fig) as being the ADLs. Recently, others have identified SKN-1B in AIY neurons as a regulator of chemosensory processes and behaviour, showing that animals lacking the skn-1a, c and b do not chemotax towards NaCl, butanone or temperature, or move towards thicker bacterial lawns (as a WT worms would) [57]. We tested our skn-1b specific mutant in a NaCl chemotaxis assay and got similar results but have no evidence that SKN-1B is endogenously expressed in the AIYs in fed conditions. It is possible that SKN-1B signals from the ASI—AIY neurons to medi [file pgen.1009358.s008.tif]

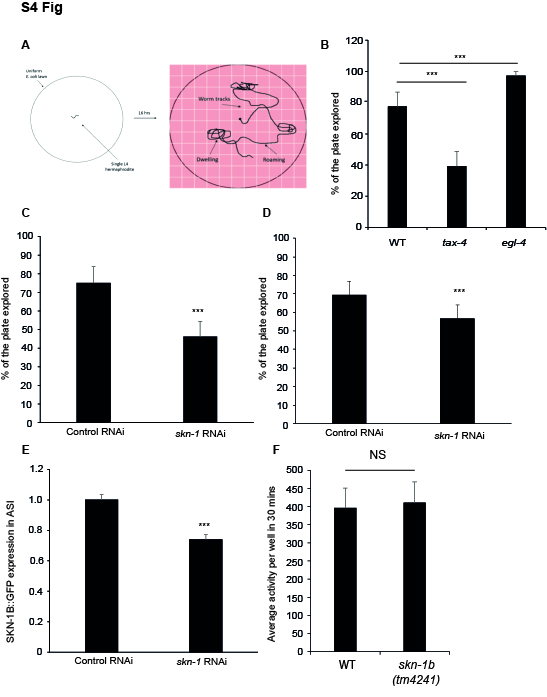

Supplement: S4 Fig — A) Cartoon showing setup of exploration assay as in [18]. Extended dwelling or roaming compared to WT behaviour can be quantified by counting the number of squares that a worm traverses over 16hrs. Food is spread evenly and continuously on the plate. B) Control experiments for exploration assay. Time spent in roaming and dwelling states depends on integrating internal neuro-modulatory cues with external sensory cues. The absence of such sensory transduction leads to extended dwelling as observed in the tax-4 mutant. tax-4 encodes a cyclic nucleotide-gated channel subunit, in contrast, mutants with constitutive sensory input, such as the egl-4, which encodes a molecule with cGMP protein kinase activity, exhibit extended roaming [73]. Representative experiment of 3 biological replicates shown, n<15 worms per group ± st. dev. C and D) Quantification of exploratory behaviour in response to skn-1 RNAi fed at either the L1 (C) or L4 stage (D). Mean plate coverage of n>23 individual worms per group ± st. dev., one representative experiment of 3 biological replicates shown. For B-D) Two-tailed t-test NS non-significant. Two-tailed t-test *p<0.05, **p<0.001, ***p<0.0001, NS not significant. E) Neurons are relatively resistant to RNAi (Timmons et al., 2001), but quantitative fluorescence microscopy shows that skn-1 RNAi from the L1 stage reduces SKN-1B::GFP in ASIs by ~30%. The smaller difference between exploration in WT and skn-1b mutants in (D) likely reflects the RNAi knock-down from L4 being less complete. The skn-1 RNAi clone used in C-E targets all skn-1 isoforms [16]. Pooled data from 3 biological replicates shown, n>100 individual worms per group. Two-tailed t-test ***p<0.0001. F) skn-1b mutants display normal thrashing activity in liquid. Average of 3 biological replicates shown, n>33 individual worms per group. (TIF) [file pgen.1009358.s009.tif]

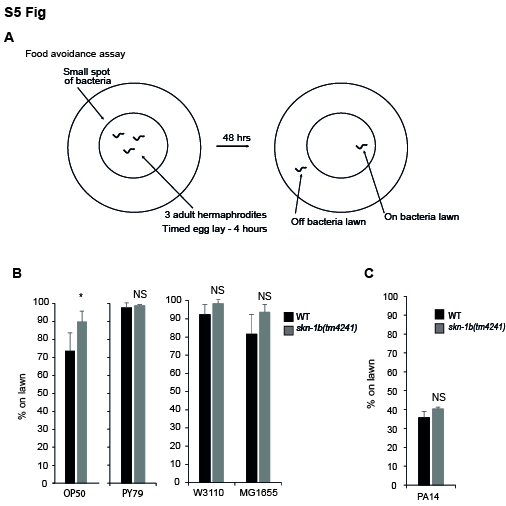

Supplement: S5 Fig — A) Cartoon showing setup of food avoidance assay as in [70]. The percentage of worms on a lawn of bacteria is determined in conditions where worms have a choice whether to be on or off the lawn. B-C) Quantification of worms on different bacterial lawns (if given a choice to leave). Other strains tested are shown in Fig 1I. Each bar represents a mean of 3 biological replicates with ~100 worms per trial ± st. dev. Two-tailed t-test *p<0.05, **p< 0.001, ***p<0.0001, NS not significant. For B-C) bacteria were allowed to proliferate in each case, and no antibiotics or FUDR were present (see Methods). NB: Our assay measures satiety quiescence following fasting, as this offers an easily quantifiable behaviour. However, satiety quiescence also occurs cyclically between foraging and dwelling when worms are fully fed. During our studies we observed that while skn-1b mutants always preferred the bacterial lawn regardless of the food type, WT worms could be tempted to spend more time on certain bacteria (Fig 1I). A similar defect in food sensing behaviour was also observed for skn-1 mutants [57]. (TIF) [file pgen.1009358.s010.tif]

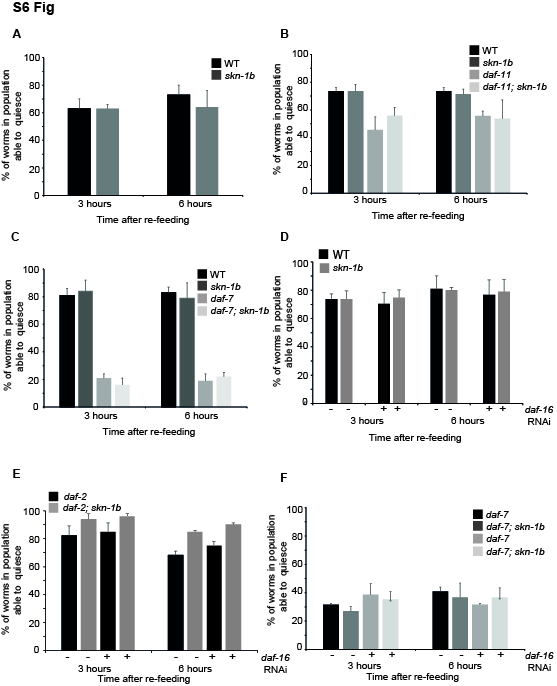

Supplement: S6 Fig — A-F) % worms spending time in quiescence 3 or 6hrs after fasting and re-feeding. Each bar represents a mean of 3 biological replicates ± SEM with n>36 worms per group. Due to the nature of the assay, satiety quiescence is not observable in every worm in an experiment, particularly in mutant strains that exhibit low levels of quiescence such as daf-7 and daf-11. Similar numbers of worms from WT and mutants were observed in quiescence (S6C Fig) but fewer daf-7 and daf-7;skn-1b mutants entered quiescence (S6C Fig). Thus, the daf-7 data in Fig 4A is likely to be an over-representation of the actual level of satiety quiescence within the population. (TIF) [file pgen.1009358.s011.tif]

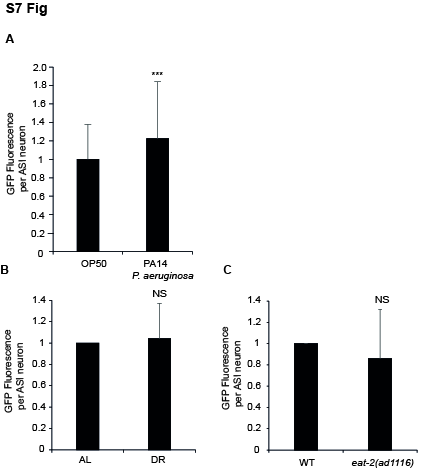

Supplement: S7 Fig — A-C) Quantitative fluorescence microscopy of SKN-1B::GFP expression in response to pathogenic bacteria (A), an alternative DR protocol (Moroz et al 2014) (B), or eat-2 mutation (C). For (A and C) bacteria were allowed to proliferate in each case, in B) antibiotics were present (see Methods). For A-C) Error bars show st. dev. Two-tailed t-test compared to day 1 expression levels *p<0.05, **p< 0.001, ***p<0.0001, NS not significant. (TIF) [file pgen.1009358.s012.tif]

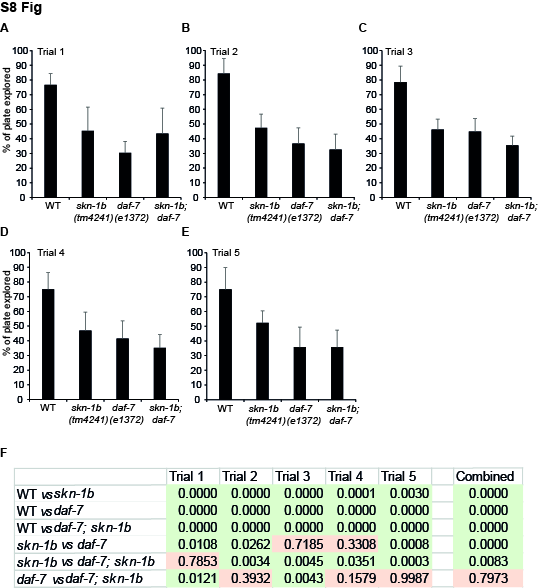

Supplement: S8 Fig — A-E) Individual exploration assays combined in Fig 4D. We reasoned that if each of the two genes regulate different behaviours independently, then the effects of daf-7 and skn-1b on behaviour should be additive. However, the exploration of daf-7 and daf-7; skn-1b worms was not significantly changed in 4 out of 5 trials i.e. not additive effect. In each experiment the mean plate coverage of n>8 individual worms per group is shown ± standard deviation. Two-tailed t-test *p<0.05, **p< 0.001, ***p<0.0001, NS not significant. F) Statistical analysis of each individual and combined trial(s). Comparisons highlighted in green are significant (two-tailed t-test p<0.05), and those in orange are NS. (TIF) [file pgen.1009358.s013.tif]

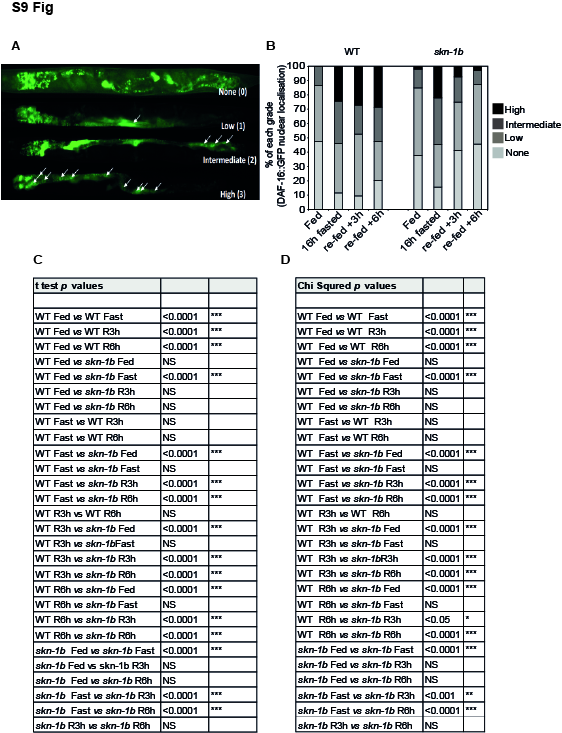

Supplement: S9 Fig — A) Scoring system for DAF-16a::GFP nuclear localisation in the gut nuclei. Nuclear localisation was graded by a four-point system; 0 = none, 1 = low, 2 = intermediate, 3 = high. Nuclear grading was carried out by a combination of the quantity of punctate gut nuclei as well as the fluorescence intensity of these nuclei. B) Quantification of the grading of the DAF-16a::GFP nuclear localisation in both WT and skn-1b mutants under fed, fasted, fasted/re-fed for 3hrs or 6hrs. C) Full statistical analysis using two tailed t-test for the average grading of DAF-16a::GFP nuclear localisation shown in Fig 5A. D) Full statistical analysis determined by chi-squared test of DAF-16 nuclear localisation data shown in S9B Fig. (TIF) [file pgen.1009358.s014.tif]

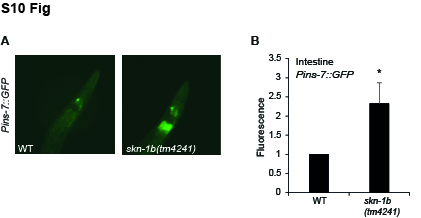

Supplement: S10 Fig — A) Representative images showing Pins-7::GFP expression in WT and skn-1b mutants. Expression was visible in various neurons and the gut. 20x magnification. B) Quantitative fluorescence microscopy of Pins-7::GFP in the gut. Neuronal Pins-7::GFP levels were not quantified as its expression in multiple neurons made their individual identification difficult. skn-1 has also been shown to repress ins-7 expression [35], consistent with the increase in ins-7::GFP observed in skn-1b animals. (TIF) [file pgen.1009358.s015.tif]

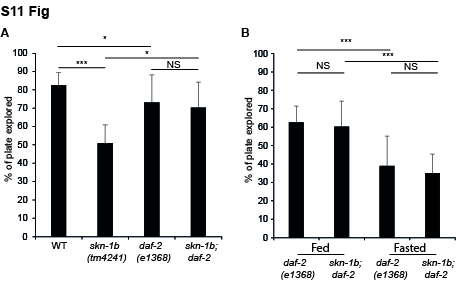

Supplement: S11 Fig — A and B) Quantification of exploration. One representative experiment of 3 similar biological replicates shown ± st. dev., n>10 worms per group. Two-tailed t-test *p<0.05, **p< 0.001, ***p<0.0001, NS not significant. daf-2(e1368) caused a milder exploratory defect than daf-2(e1370) (Fig 5B). Fasting also reduced exploration in daf-2(e1368) animals but this was not further reduced by skn-1b mutation (Fig 5C). Therefore, despite this milder daf-2(e1368) exploratory phenotype, skn-1b mutation was not able to further suppress exploration in either fed, or fasted and re-fed conditions. (TIF) [file pgen.1009358.s016.tif]

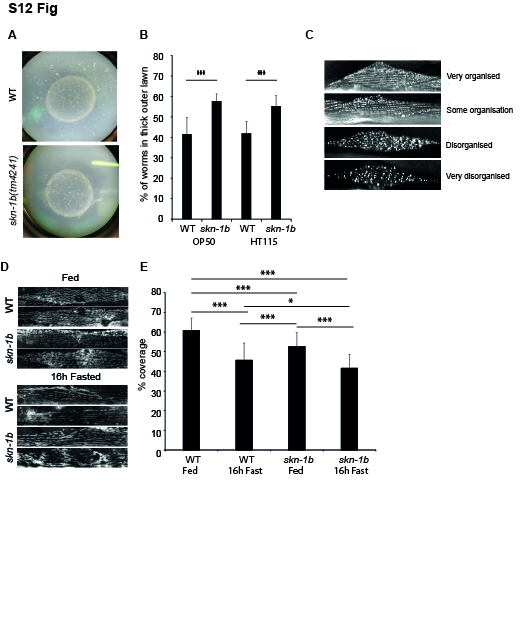

Supplement: S12 Fig — A and B) Images and quantification of bordering behaviour. Each bar represents a mean of 3 biological replicates ± st. dev. C) Scoring system of the expression of myo-3::mitoGFP in C. elegans. D and E) Expression and quantification of WT and skn-1b mutant C. elegans expressing tomm20:GFP. This reporter expresses a peptide of tomm20, an outer mitochondrial membrane protein and hence marks all mitochondria, delineating their shape [39]. In E) Each bar represents a mean of 3 biological replicates ± SEM, n>49 day 1 adults worms per group. Note that although fluorescence microscopy demonstrates an alteration in the organisation of the mitochondrial networks and suggests a level of disruption, it is comparison with TEM (Figs 6D, S12, S13 and S14) that allowed us to define the nature of the disruption. (TIF) [file pgen.1009358.s017.tif]

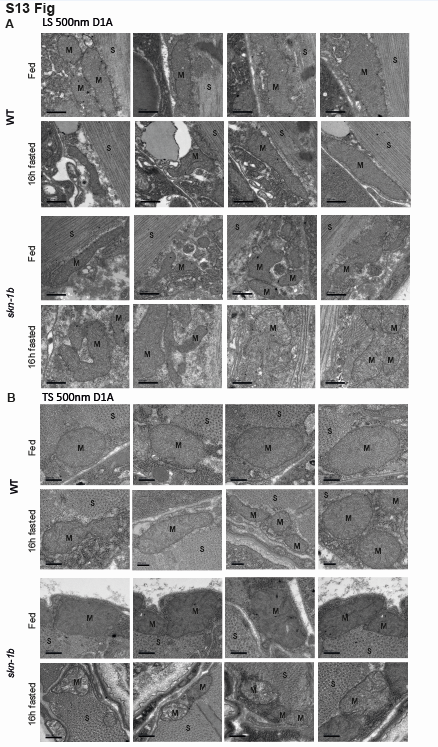

Supplement: S13 Fig — A) Longitudinal sections and B) Transverse sections. All scale bars = 500nm, M = mitochondria, S = sarcomere. Fasting disrupts mitochondrial networks in response to fasting in WT animals. skn-1b mutants also have disrupted mitochondrial networks, exhibiting increased fusion of mitochondria. In response to fasting skn-1b mutant mitochondria appear much worse than WT, with disrupted membranes and cristae structures. (TIF) [file pgen.1009358.s018.tif]

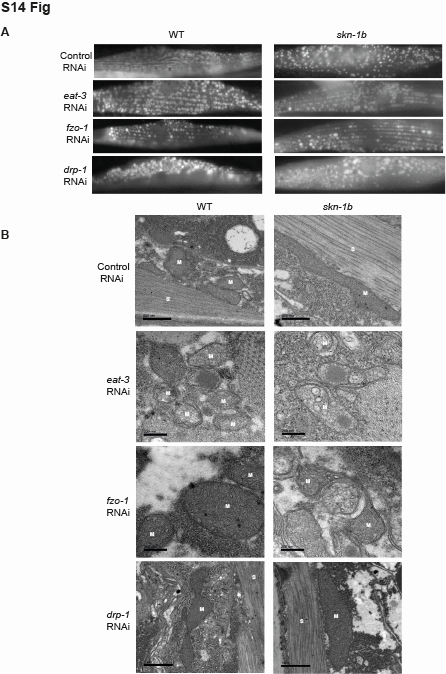

Supplement: S14 Fig — Images of myo-3::mitoGFP (A) and TEM images (B) in WT and skn-1b mutant C. elegans fed control, eat-3, fzo-1 or drp-1 RNAi. TEM shows Longitudinal sections 200nm. Note that although the fluorescent images in A clearly show signs of mitochondrial network disruption, it is only when examining the TEM images that the precise network structures can be seen e.g. both eat-3 and drp-1 RNAi show a “spotty” pattern on the fluorescent images but this translates to a very different TEM image with eat-3 RNAi causing fission and drp-1 RNAi fusion (as expected). (TIF) [file pgen.1009358.s019.tif]
