# Supplementary material for: Neuronal SKN-1B modulates nutritional signalling pathways and mitochondrial networks to control satiety
Source: PLoS Genet. 2021 Mar 4;17(3):e1009358. doi: 10.1371/journal.pgen.1009358 (PMC7932105; doi:10.1371/journal.pgen.1009358)
Supplement: Attachment — Submitted filename: 109208_1_rebuttal_1889606_qd8qgt.docx [file pgen.1009358.s020.docx]

**Response to reviewers/ Review Plan**
------------------------------------------------------------------------------
**Reviewer #1 (Evidence, reproducibility and clarity (Required)):**
*This paper is very comprehensive and the data presented in the figures shows significant changes in behavioral measures. Each figure shows an experiment that most will find convincing. I suggest that the paper be published AS IS. It is comprehensive and thorough. And well done. Congratulations to the authors. A note to the other reviewers and to the authors: my reviewing of papers seeks to change the system of review by example. Most papers that I am sent for review are 95% perfect. If I comment on the 5% that could lead to a small improvement, it slows down the publication of the 95% that is very valuable. So it is not in the interest of the scientific enterprise to slow down publication of all papers, often by a year, for 5% improvements. I think that this is true for almost all reviewing. Of course there is an underbelly in scientific publishing of terrible science and fraudulent science that needs reviewing to be excluded from polluting the generally excellent science that is published. So I agree that reviewing is needed. But this paper is in the top tier that should be published AS IS.

Reviewer #1 (Significance (Required)):
There is much to recommend in publishing this paper. There is a large audience who are interested in the skn-1 transcriptional network and the relationship to the ASI neuron endocrine outputs is also generally interesting to a wide audience.*
We thank the reviewer for their very positive response to our study and are pleased that they found the data convincing. We hope that by using this open reviewing platform, we are contributing to positive change in the review system.

--------------------------------------------------------------------------------------------------------------------------------------
**Reviewer #2 (Evidence, reproducibility and clarity (Required)):**
*The manuscript by Tataridas-Pallas et al. reports that the ASI-expressed SKN-1B isoform controls food-related behaviors in C. elegans that have been likened to satiety in mammals. However, unlike the skn-1a and c isoforms, skn-1b is reported to be non-essential for dietary restriction-mediated lifespan extension. Sensory perception via daf-11 is required upstream of skn-1b, which modulates TGFβ and insulin signaling downstream. Further, while skn-1b mutant animals showed signs of being over-fed compared to WT animals due to these behavioral differences, the mitochondrial networks in muscle cells of fed skn-1b mutants appeared more similar to WT fasted animals. The authors conclude that skn-1b is a central signaling node that responds to food cues, and that skn-1b mutant animals' inability to appropriately perceive their well-fed state results in a starvation state at the physiological and behavioral levels.*

*I found this paper very interesting and that the experimental evidence sufficiently supported their claims. However, I do have some minor comments to improve the clarity of some experiments and results.*
We are extremely pleased that the reviewer shares our enthusiasm for this study and that they find the experimental evidence compelling. We can address their minor comments by carrying out a small number of simple, defined experiments. Discussed below.

***1.*** *Because there are so many different ways to control the amount of food in the environment and is related to such a crucial result, I would have found a brief description of the dietary restriction protocols used and the main differences helpful, whether in the methods or the body of the results, rather than just citing other papers.*
We agree that this would be helpful. We will add a table to the methods section which details each DR protocol and their relationship to *skn-1*.

***2.*** *I think it would be useful to measure quiescence in WT and skn-1b mutants under fed conditions. The authors show that skn-1b mutants explore the plate much less than WT (Fig. 2A) and are larger than WT animals (Fig. 2E), leading one to think this must be due to skn-1b mutants eating more while on the lawn. However, after fasting (Fig. 2C), skn-1b mutants actually spend much of that time in quiescence and not eating. The phenotypes seen in the animal size (Fig. 2E) and food ingestion (Fig. 2F) experiments seem quite large given the modest effects skn-1b has on pumping. Perhaps an extra figure panel showing pumping period would also be helpful to add more context to the pumping data in Fig. 2D.*

Thank you for suggesting this experiment, we agree that it would enhance our study. We report that an absence of *skn-1b* caused strikingly reduced exploratory behaviour (in fed conditions), which could be explained by increases in quiescence, particularly as we observe *skn-1b* mutants to exhibit increased satiety induced quiescence in response to fasting and refeeding. To expand this and determine the precise behaviours underlying the decreased exploration we will measure and compare the time spent in all three *C. elegans* behavioural states (roaming, dwelling and quiescence) in fully fed WT and *skn-1b* mutants. This behavioural profile will be generated using our recently acquired WormTracker (MBF Bioscience). This experiment will tell us accurately how the different food related behaviours integrate with the other food related parameters we have measured (worm size, pharyngeal pumping and *E, coli* accumulation in the gut) in both fed and fasted: refed conditions.

***3****.The data in Fig. 5D could use some clarification. The skn-1b;daf-2;daf-16 triple mutant was more quiescent than the skn-1b;daf-2 double mutant, despite the fact that daf-16 is required for the increased quiescence of daf-2 mutants. This could indicate that there is an unknown factor that becomes important only in the absence of skn-1b and daf-16. However, given that TGFβ is known to regulate insulin signaling (Greer et al. 2008, Shaw et al. 2007) and quiescence, perhaps this data is bringing attention to the interaction of these two pathways. In other words, skn-1b could be directly regulating the expression of DAF-7/TGFβ, which then is what is actually responsible for regulating insulin signaling. This would be fairly easy to test by looking at various double mutants between these pathways.*
This is an incredibly interesting point, and we thank the reviewer for raising it. Our data so far suggests that SKN-1B acts upstream of both IIS and TGFβ to control behaviour, placing it as a new central node in the ASI behavioural response pathways. The suggested relationship between TGFβ and IIS is certainly possible. Increased nuclear localisation of DAF-16 is observed in *daf-7* mutants (Shaw et al 2007) however, this does not marry with the idea that *daf-7* mutants do not quiesce (Gallagher et al., 2013; You et al., 2008; Figure 4A), and that *daf-7* suppresses the quiescence of *skn-1b* mutants (Figure 4A). However as SKN-1B is acting so centrally, it is possible that in its absence this relationship alters. To test this we will carry out a straightforward satiety induced quiescence assay in *daf-7* and *daf-7; skn-1b* mutants in the presence and absence of *daf-16* RNAi. We already know that *daf-16* RNAi is effective at suppressing the increase quiescence of *daf-2* mutants (similarly to the *daf-16* mutant), showing that this genetic strategy works effectively in this assay (preliminary data, not shown). This experiment will identify whether or not IIS and TGFβ interact to control quiescence downstream of *skn-1b*. The experiment poses no risk and the outcome will be informative either way.

*Reviewer #2 (Significance (Required)):
This manuscript represents a conceptual advance in the understanding of SKN-1/Nrf function, satiety quiescence regulation, and fills in a gap between sensory perception and the resulting endocrine signaling. The authors find a new function of SKN-1/Nrf as a central node to regulate endocrine signaling responses to environmental food cues. Further, this function is specific to a single isoform, skn-1b, and is independent of dietary restriction-mediated longevity, which had previously been the main link between SKN-1 activity and metabolism (Bishop and Guarente, 2007). This paper would be of interest broadly to anyone studying how sensory perception affects behavior, endocrine signaling, and mitochondrial networks. My expertise lies in C. elegans, aging, and insulin signaling.
Bishop N a, Guarente L. 2007. Two neurons mediate diet-restriction-induced longevity in C. elegans. Nature 447:545-549. doi:10.1038/nature05904*
We fully appreciate the recognition that our data provides a conceptual advance in how we view sensory perception and endocrine signalling, as well as identifying a novel function of SKN-1/Nrfs. As the reviewer points out, all past work has focused on SKN-1B and dietary restriction but this study demonstrates that SKN-1B has much broader metabolic roles. We agree that this paper would be of interest to a broad audience i.e. those from metabolic, behavioural, endocrine, mitochondrial, and ageing biology backgrounds, and that it suggests novel metabolic functions of mammalian Nrf proteins.

***Reviewer #3 (Evidence, reproducibility and clarity (Required)):***
*Tataridas-Pallas and Thompson et al. describe a role for the ASI-expressed SKN-1 isoform SKN-1B in mediating food sensing and food-related behaviour in C. elegans. The authors show that SKN-1B modulates satiety quiescence by supressing DAF-7 expression within the ASI neurons, which alters downstream TGF-beta signalling. SKN-1B affects the morphology of mitochondria in distal tissues, which modulates the behavioural feeding state of the animal in response to altered food conditions.*

***Major comments:**
*Are the key conclusions convincing?**
***1.*** *Although it has been previously shown that skn-1 plays a role in DR-longevity, the statement that "skn-1b contributes to, but is not essential for DR longevity" is not convincing based on the presented data. The eat-2 genetic model is a complex model for dietary restriction (for example PMID: 30965033), and skn-1b was required for longevity enhancement only for the genetic (eat-2) DR model, and not in the bacterial-dilution DR model used. Given that, among other food-sensing/eating phenotypes, eat-2 mutants have reduced pharyngeal pumping, and the evidence that follows in the manuscript show that skn-1b mutants have increased pharyngeal pumping/food intake, these data suggest that eat-2; skn-1b double mutants are likely to have wild-type food intake, i.e. are not dietary restricted at all. Thus, it cannot be concluded that skn-1b has any effect on the longevity by dietary restriction but affect the efficacy of using the eat-2 mutant as a genetic model for DR-longevity itself.*

- This is an interesting point. To be clear, in our statement "*skn-1b* contributes to, but is not essential for DR longevity" we intended to refer not only to the *eat-2* and bacterial dilution experiments in Figures 1A-B, but also to the bacterial dilution experiment previously published by the Guarente lab (Bishop and Guarente 2007). In this 2007 work, they showed that a *skn-1(zu135)* mutant (which lacks all *skn-1* isoforms) did not exhibit an increased lifespan in response to DR. However, the DR longevity response was rescued by expressing *skn-1b* specifically in the ASI neurons. Thus, in that scenario *skn-1b* did indeed contribute to DR lifespan. This paper was cited in this section of the manuscript, but we will also add it following this statement to increase clarity.
- We agree that the *eat-2* DR model is complex and to address the issue of pumping rate in *eat-2; skn-1b* double mutants confounding its DR potential, we will measure and compare pumping rate in *eat-2 vs eat-2; skn-1b* animals. NB: When the *eat-2; skn-1b* double mutant was created, it was genotyped for *skn-1b* using PCR and for *eat-2* by a phenotype approach i.e. worms were identified with slow pumping and skinny/pale appearance. Therefore, we feel that it is not likely that *skn-1b* mutation will dramatically affect the *eat-2* pumping but our proposed analysis will check for any subtle differences.

***2.*** *That skn-1b is regulating exploration behavior, and acts in the same genetic pathway as the gyanylate-cyclase daf-11 in exploration behaviour is convincing. As is the data showing the skn-1b and daf-11 regulate the appropriate exploration behavioural response to fasting and refeeding. DAF-11 enables ASIs to sense the external environment, and is required for the skn-1b expression response to fasting, however how daf-11 alters skn-1b levels, given their different subcellular locations is unclear. The data clearly show that DAF-7 was required for the increased satiety quiescence in the skn-1b mutant, and loss of skn-1b leads to increased daf-7 expression in the ASI neurons. However, the opposing roles of daf-7 and skn-1b in quiescence are not applicable to the exploration behaviour, where daf-7 and skn-1b appear to both promote exploration. Given that daf-11 is required for daf-7 expression in the ASI (PMID: 11677050), placing this ASI gene regulatory program into more context may make these findings more convincing and clear.*

- Thank you for this insightful comment and we are glad you find our data relating to *skn-1b* and *daf-11* exploratory behaviour convincing. We believe that this nicely links environmental food status with the main body of the ASI neuron. Understanding how *daf-11* ultimately impacts on *skn-1b* activity is fascinating but beyond the scope of this study. The behavioural aspects of these molecular interactions are highly testable though and boil down to differences in behaviour in response to different food conditions. In fasted: refed conditions *daf-11* and *daf-7* are required for satiety induced quiescence (Gallagher et al., 2013) and here we show that *skn-1b* suppresses satiety induced quiescence. Interestingly though, fully fed *daf-7* mutants spend more time dwelling (Gallagher et al., 2013). Therefore, we will focus on quantifying the behaviour profile (roaming, dwelling and quiescence) of *skn-1b* and *daf-11* mutants (compared to WT) in fed conditions using our newly acquired WormTracker (also see our response to reviewer 2, comment 2). This simple experiment will fill the gaps in the knowledge and fully define the behavioural roles in relation to food (fed *vs* fasted; refed) for each of these three key ASI expressed genes.
- If necessary, depending on the outcome of this experiment, we can expand this to examine the behavioural profile of our *daf-7; skn-1b* and *daf-11; skn-1b* double mutants. This would determine the epistatic interaction of *skn-1b* with *daf-7* and *daf-11* respectively in fed conditions.

***3.*** *The data and conclusions about SKN-1B's interaction with insulin-like signalling is unconvincing and requires some further experiments and explanation. DAF-16 and DAF-2 appear to act in different genetic pathways. In our experience, an additive effect with the daf-2(e1370) mutant in exploration assays is impossible to measure, as this mutant explores so little, less exploration is unquantifiable. Indeed, there may be an additive effect in the daf-2;skn-1b double mutant, which is non-significant simply because of the limitations of this assay. Supporting this is that there is clearly an additive effect of skn-1b; daf-2 mutant in quiescence duration, indicating that skn-1B acts in a different pathway to insulin-like signalling. As the experiment of daf-16 RNAi on skn-1b quiescence duration alone was not performed, the data presented in Fig 5D are not fully interpretable. Any effect of skn-1b on IIS may be a "side effect" of skn-1b's role in food consumption and not a result of direct signalling to this pathway.*
Thank you for this comment. However, we believe that our data does show that *skn-1b* interacts with the Insulin/*daf-2* pathway: To control DAF-16 nuclear localisation; exploration behaviour; and satiety induced quiescence. This interaction may not be linear in every case, and may vary depending on food status but it is definitely there. To address this further we propose examining the IIS/*skn-1b*/behaviour relationship for each food condition separately.

- Exploratory behaviour in fed conditions: We show that *skn-1b* cannot further suppress the exploration deficiency of two different *daf-2* alleles, *daf-2(e1370)* (Figure 5B WT *vs daf-2(e1370)* *p*=0.0001); *daf-2 vs daf-2; skn-1b p*=NS)) and *daf-2(e1368)* (Figure S11 (WT *vs daf-2(e1368)* *p*=0.0001); *daf-2 vs daf-2; skn-1b p*=NS) mutants. The reviewer suggests that it would be challenging to further suppress (the low) exploration of *daf-2(e1370)* mutant, but by also showing the same effect using a milder *daf-2(e1368)* allele, which exhibits an intermediate reduction in exploration, our argument is stronger. Overall, we believe that SKN-1B and IIS do act in the same pathway to control exploration in fed conditions.
- Exploratory behaviour in fasted: refed conditions: Similarly to the fed scenario, we found that *skn-1b* could not further suppress the *daf-2(e1370)* fasted: re-fed exploration phenotype. As above, the reviewer suggests that it would be challenging to further suppress (the low) exploration of *daf-2(e1370)* mutant in these conditions. Therefore, we will repeat this experiment using the milder *e1368* allele. We predict that fasting and re-feeding will cause a further reduction in *daf-2(e1368)* exploration, but as the effects are likely to be more intermediate, this experiment will determine the impact of *skn-1b* mutation on exploratory behaviour. This will confirm whether IIS and *skn-1b* interact epistatically in relation to exploratory behaviour in fasted: refed conditions.
- Satiety induced quiescence behaviour: The reviewer notes that an additive quiescence effect is seen between *daf-2* and *skn-1b* mutations following fasting and refeeding. That could imply that the two act independently. However, while the key transcriptional mediator of IIS (*daf-16)* is required for the high quiescence of *daf-2* mutants, in the absence of *skn-1b* this effect is lost. Taken together with the information that *skn-1b* is required for *daf-16* to be maintained in intestinal nuclei we interpret this as suggesting that IIS and *skn-1b* interact but that additional factors are required. As it stands our data set does not allow the effects of *daf-16* on WT and *skn-1b* quiescence to be compared with the situation in a (reduced IIS) *daf-2* background. Therefore we will do an additional control experiment comparing WT and *skn-1b* mutants in the presence and absence of *daf-16 RNAi*. We know that *daf-16* RNAi will work for this assay (preliminary data, not shown). This will determine the behavioural interactions of *daf-16 and skn-1b* in both normal and reduced IIS conditions.

***4.*** *The data on mitochondrial networks are unclear. The fluorescence microscopy analysis demonstrates that skn-1b mutant has a high proportion of fragmented and very fragmented mitochondria compared to wild-type, which is exacerbated with fasting. However, the electron microscopy data shows that skn-1b mutants exhibit a more fused-like state than wild type. It is unclear what the explanation is for these opposing results.*
Thank you for raising this. We believe that theconcept of the neuroendocrine system and *skn-1b*/Nrf impacting on mitochondrial dynamics and ultimately behaviour is a unique and fascinating aspect of our story. Conceptually however, the reviewer has hit on a technical point that should be made to a wide audience; that direct comparison of fluorescent and TEM images do not always match. We do not believe that our results are opposing, but agree that we could add further information to support this. To address this we will:

- Prioritise expanding our genetic experiments that specifically disrupt mitochondrial fission or fusion. Specifically, we will include data from animals treated with *eat-3*, *fzo-1* and *drp-1* RNAi (causing increased mitochondrial fission, fission and fusion respectively). By using our strategy of comparing fluorescent and TEM images our data will demonstrate the mitochondrial network patterns observed when known mitochondrial network proteins are disrupted.
- Expand our data set examining the link between *skn-1b*, mitochondrial dynamics and behaviour. We show that *eat-3* RNAi (causing mitochondrial fission) is sufficient to reverse the *skn-1b* reduced exploration phenotype and will next use our genetic tools to test the effect of both mitochondrial fission and fusion on *skn-1b* behaviour. This can be easily achieved by feeding WT or *skn-1b* mutants with *eat-3, fzo-1* and *drp-1* RNAi prior to measuring their exploration (similarly to data already in Figure 6F-H). These data will complete the picture of how *skn-1b* interacts with mitochondrial dynamics to control behaviour. Again, we will also show fluorescent and TEM data to clearly demonstrate the effect on the mitochondrial network for each genetic intervention.
- Alter the language in Figure 6C which shows a more qualitative analysis of this mitochondrial disruption (determined by examining fluorescent images and grading them). The key will now read, “Very disrupted, Disrupted, Mild disruption and No disruption”. This does not alter the data presented but removes assumptions about the precise nature of the mitochondrial networks which could be construed as misleading.

**Should the authors qualify some of their claims as preliminary or speculative, or remove them altogether?**
***1.*** *The leaving assay on different bacteria (fig1I) is insufficient to show that skn-1b acts to sense food types, this would require testing whether skn-1b chooses one bacteria over another in a food choice assay (see PMC1352325). Instead the data show that skn-1b mutants behave in a satiated manner, regardless of the "quality" of the bacteria.*

To address this we will alter the text from *“As almost all skn-1b mutants are present on an OP50 lawn, it implies that while WT worms adapt to preferentially consume some foods, skn-1b mutants do not”* to; *“As almost all skn-1b mutants are present on an OP50 lawn, it implies that they are behaving in a satiated manner”.*

***2.*** *The data forming the conclusion that SKN-1B::GFP expression responds to different bacterial types and dilutions, and responds selectively to the amount of food available requires more explanation and interpretation. As the two bacteria (PY79 and PA14) have the opposite effect on the behaviour, and that skn-1B was not required for sensing and avoiding PA14, please clarify how the skn-1b expression level is reconciled with opposite outputs in behaviour.*

This is an interesting point. The transcriptional outputs of other SKN-1 isoforms are known to differ depending on the stimulus e.g both *tert*-butyl hydroperoxide and arsonate are known to cause SKN-1C::GFP translocation to intestinal nuclei but result in completely different transcriptional outputs (Olivera et al., 2009 PMID: 19575768). It is possible that this is also the case for SKN-1B. Alternatively, in the case of the pathogen response, perhaps additional immune signals are sufficient to override any satiety behaviour in the *skn-1b* mutants? This will be added to the discussion.

***3.*** *Loss of SKN-1B does not affect DAF-16 localisation in either fasted or fed conditions, but was required for DAF-16::GFP to be maintained in the nucleus after fasting/refeeding. Could this be a downstream effect of the increased feeding behaviour of the skn-1b mutant? Given that skn-1b mutants have a much higher food intake and pumping rate, does this lead to a "satiated/fed" metabolic state much faster than in wild-type worms? If the skn-1b mutant recovers from fasting far more quickly than wild-type worms, does this then lead to a fast recovery of daf-16 localisation?*
We know that *skn-1b* mutants spend longer in quiescence following fasting and refeeding but we don’t know if this equates to *skn-1b* mutants recovering faster than WTs from the fasting. The *skn-1b* mutants only have a very moderate increase in body size, gut *E. coli* and pumping rate ~10% under these conditions, whereas *daf-16* mutants demonstrate ~50% increase in food consumption e.g. Wu et al., 2019 PMID: 30905669. If the assumption is that DAF-16 acts to limit feeding then its seems counterintuitive that it would be “lost” in the *skn-1b* mutants where it could potentially be considered more important to limit food intake. There are also examples of mutants where food intake is increased e.g. *daf-7* mutants (Gallagher et al., 2013) but where quiescence is not possible meaning that the two processes can be dissociated.

*Would additional experiments be essential to support the claims of the paper? Request additional experiments only where necessary for the paper as it is, and do not ask authors to open new lines of experimentation.*
***1.*** *Do eat-2;skn-1b double mutants have WT pharyngeal pumping/food intake? This simple experiment would identify whether or not there should be DR-lifespan extension. If the double mutants eat the same amount of food as WT this is not a dietary-restricted model. To reconcile these findings with Bishop and Guarente (2007), which uses the liquid DR protocol that induces skn-1b expression. The same DR protocol as used by Bishop and Guarente (2007) with the skn-1b-specific mutant allele would be useful. Using the liquid DR and an additional (different, i.e chemically defined) DR protocol to determine whether skn-1b (specifically) contributes to lifespan extension under dietary restriction in any other specific cases.*

- We agree that the *eat-2* DR model is complex and to address the issue of pumping rate in *eat-2; skn-1b* double mutants confounding its DR potential, we will measure and compare pumping rate in *eat-2 vs eat-2; skn-1b* animals. NB: When the *eat-2; skn-1b* double mutant was created, it was genotyped for *skn-1b* using PCR and for *eat-2* by a phenotype approach i.e. worms were identified with slow pumping and skinny/pale appearance. Therefore, we feel that it is not likely that *skn-1b* mutation will dramatically affect the *eat-2* pumping but this analysis will check for any subtle differences.
- Our data show that SKN-1B is not generally essential for DR (when comparing two protocols in our m/s with one published from the Guarente lab) so we are doubtful that testing multiple other protocols would be informative. In *C. elegans*, DR protocols give vastly different lifespan extensions and there is even more variability between labs. It is possible that the % extension in lifespan incurred by a DR protocol affects the ability of SKN-1B to contribute to it i.e. both the Guarente DR and *eat-2* are fairly modest extensions whilst the Moroz DR extension is huge. Defining this further would be very challenging, and to paraphrase the reviewers own words below, “very time consuming and not essential for publication”. The main focus of the manuscript is our work examining the novel role of SKN-1B in food related behaviour.

***2.*** *SKN-1B and DAF-11 act in the same genetic pathway to regulate exploration behavior. SKN-1B acts to inhibit satiety quiescence and pharyngeal pumping after fasting, while previous data (in discussion, You et al 2008) show that daf-11 promotes satiety quiescence after fasting/refeeding. How does this tie in with exploration, as increased quiescence is the reason why skn-1B mutants do not explore? Performing the quiescence-duration assay in the daf-11, daf-11;skn-1b strains would aid in clarifying whether daf-11 or skn-1b has the dominant role in regulating pharyngeal pumping.*

We agree that these are interesting questions and have already discussed (above) the interesting interplay between *skn-1b, daf-11* and *daf-7* in a single pair of neurons (the ASIs). To address this we will quantify the behavioural profile (roaming, dwelling and quiescence) of *skn-1b* and *daf-11* mutants in fed conditions using the Wormtracker (MBT). This straighforward experiment will provide new behavioural information on both genes and tell us whether *skn-1b* and *daf-11* mutants behave similarly or differently in fed conditions (in contrast to their opposite roles in satiety induced quiescence). Together with published data, it will also complete the picture of how each ASI gene impacts on behaviour in response to different food conditions. Depending on the outcome of this experiment, we can also easily expand it to test the behaviours of our *daf-11; skn-1b* and *daf-7; skn-1b* double mutants in fed conditions so that the epistatic relationship of the two genes can be determined.

***3.*** *The IIS quiescence measurements presented in Figure 5D would benefit by having the wild-type control for comparison, but requires skn-1b alone and with daf-16 RNAi to be able to interpret these data appropriately.*
We will add in four additional controls: WT, *skn-1b*, WT *daf-16* RNAi and *skn-1b daf-16* RNAi to this data set.

***4.*** *The eat-3 RNAi rescue of skn-1b exploration shows that inhibiting mitochondrial fusion rescues the skn-1b phenotype, given that the fluorescence microscopy assay showed the opposite effect, then performing a couple of control experiments may help unravel these opposing results: Does the eat-3 RNAi lead to increased fragmentation in the mito::GFP assay? Also, does drp-1 RNAi (preventing fission) have any affect on skn-1b behaviour and mitochondrial morphology?*
As discussed in detail above, we will prioritise a set of genetic experiments to fully define the roles of mitochondrial fission and fusion in regulating behaviour. This will be complemented by fluorescent and TEM images in each condition to clearly demonstrate what each genetic manipulation (*eat-3, fzo-1* and *drp-1* RNAi) is doing to the mitochondrial networks. Note: Our preliminary experiments show that *fzo-1* RNAi (causing fission similarly to *eat-3* RNAi) has similar behavioural effects to *eat-3* RNAi (in both WT and *skn-1b* mutants). However, *drp-1* had no behavioural phenotype (despite affecting mitochondrial network morphology). This supports our idea that mitochondrial fission promotes normal food-responsive behaviour but suggests that over-fusing mitochondria is not sufficient to alter behaviour (as also shown by Bryne et al., 2019 PMID: 30840087 ).

*Are the suggested experiments realistic in terms of time and resources? It would help if you could add an estimated cost and time investment for substantial experiments.*
*The suggested lifespan experiments would take a substantial amount of time, and I think these experiments are only essential if the authors wish to truly link the function of skn-1b to DR-longevity.
All other experiments suggested are feasible, and low cost in time and resources.*

We completely agree with the reviewers’ assessment of this situation. We will address the issue of the *eat-2* DR model (as specified above) but do not plan to take the lifespan studies further. We propose experiments to address all of the others reviewers comments.

*Are the data and the methods presented in such a way that they can be reproduced?*
*Yes, methods are very well written and sufficiently detailed. The only issue is the Pins-7::GFP is not stated (comment below).*

The strain in question is JMT51 *skn-1b(tm4241) wwEx66 [Pins-7::GFP + unc- 119(+)]*, made by crossing HT1702 *wwEx66 [Pins-7::GFP + unc- 119(+)]* with GA1058 skn-1b(tm4241). HT1702 was obtained from the CGC and has been published in Ritter AD, et al. Genome Res. 2013 Mar 28.

This will be added to the methods section.

*Are the experiments adequately replicated and statistical analysis adequate?*
*Yes, number of replications and statistics are adequate.*

**Minor comments:**
*Specific experimental issues that are easily addressable.*
*The INS-7 analysis is unclear. The strain name/number for the Pins-7::GFP reporter is not stated in the strains list and the images (S10A and S10B) look strikingly different to previously published images of Pins-7::GFP reporters (PMCID 18025456 and 3569836). Please clarify the differences in this reporter strain and why the expression pattern of this reporter looks different from previous studies.*
The strain in question is JMT51 *skn-1b(tm4241) wwEx66 [Pins-7::GFP + unc- 119(+)]*, made by crossing HT1702 *wwEx66 [Pins-7::GFP + unc- 119(+)]* with GA1058 *skn-1b(tm4241)*. HT1702 was obtained from the CGC and has been published in Ritter et al, 2013 PMID: [23539137](https://www.ncbi.nlm.nih.gov/pubmed/23539137).

This reference will be added to the methods section. In Ritter et al., there is a helpful table (S3) showing the comparison on this reporters’ expression with those from Pierce et al. 2001 PMID: [11274053](https://www.ncbi.nlm.nih.gov/pubmed/11274053) and Murphy et al. 2007 PMC2141905. There are expression differences, but the head neuron and intestinal expression are the same in all three reporters. Note that in our manuscript only *ins-7*::GFP in the intestine is measured (Figure S10B).

*Are prior studies referenced appropriately?*
***1.*** *The statement that ASI neurons express and secrete ~40 C. elegans insulin-like peptides is an error. To my knowledge, ~40 is the total number of C. elegans insulin-like peptides, and while many are expressed in the ASIs, multiple are not. If I am mistaken, include the citation(s) that support this statement.*
Our apologies, this was an error, we will correct it to read *“ C. elegans express and secrete ~40 insulin-like peptides (ILPs), at least some of which bind to the DAF-2 insulin/IGF-1-like receptor in multiple tissues (Pierce et al., 2001).”*

***2.*** *There are several concluding statements that do not refer to prior studies appropriately:
The publication PMC5411085 shows that skn-1b functions in the chemosensory interneurons, and is required for correct food-seeking behaviour - specifically, that skn-1 mutants have impaired food leaving behaviour. This should be appropriately cited in the context of describing the results in Fig 1I and S5B.*
We agree that this was an oversight, although this publication has been cited we could have done so more appropriately. We will add citations for Wilson et al., 2017 PMID: [28459841](https://www.ncbi.nlm.nih.gov/pubmed/28459841) in the figure legends for both Fig 1l and S5B.

***3.*** *Figure 4 in the publication PMID: 17538612 (Bishop) shows data examining the effect of DR on skn-1b expression that is consistent with Fig 3C. This should be appropriately cited.*
The reference will be added as follows: *“We found that diluting bacteria in liquid culture increased ASI expression of SKN-1B::GFP (Figure 3C, Bishop and Guarente 2007)”.*

***4.*** *Please cite which published work is in agreement with Figure 4A, this is mentioned in the text but the cites are not present.*
The text will be altered to read: “*In agreement with published work, WT animals showed increased quiescence following re-feeding, but daf-7 mutants did not (Figure 4A, (Gallagher et al., 2013, You et al., 2008))*”.

***5****. The publication PMID: 19575768 (Oliveira) have shown that skn-1 represses ins-7 expression, which is consistent with the observation in Figure S10 and should be cited appropriately.*
We will add the following sentence to support the data in Figure S10: *“ skn-1 has also shown to be shown to repress ins-7 expression (Olivera et al 2009), consistent with the increase in ins-7::GFP observed in skn-1b animals.”*

***6.*** *Please cite the study (or studies) that demonstrate skn-1 binding sites in ILP promoters.*
We believe this information is included: *“Some ILPs, have skn-1 binding sites in their promoters, making direct regulation by SKN-1B possible. One of these is ins-7 which is expressed in several neurons, including ASIs, and the gut (Murphy et al., 2007; Oliveira et al., 2009).”*

*Are the text and figures clear and accurate?*
***1.*** *rIIS was not defined in the text before using the acronym.*
Our apologies. The first time it is mentioned is in the first paragraph of the results: *“Like DR, rIIS extends lifespan in many species and skn-1 is known to be an important mediator of this (Ewald et al., 2014; Tullet et al., 2008).”* At this point we will change it to *“Like DR, reduced IIS (rIIS) extends lifespan…”.*

***2.*** *microscopy images of reporter genes in the ASI require improvement. Some basic alterations such as a scale bar, and ensuring all images have the worm arranged in the same direction (nose to the left). Some images are too blurry and imaged at too low magnification (not specified in methods) to support the conclusions. Figs 4B and S10A in particular would benefit from including the DIC and having at a stronger magnification (100X), to ensure that the compared images were taken at the same plane, with ASIs in focus*.
To address these points we will: add in scale bars; information on magnification; and adjust image orientation where possible. In any instance where conclusions have been drawn from microscopy images e.g. Fig 4B, the data was quantified and the fluorescence intensity of individual neurons measured.

***3.*** *Please include (in parentheses) the names used in the figures for the different bacteria beside the names in the text (and in fig legend). For example: another four E. coli strains (E. coli B, BL21G, HT115 and HB101), Comamonas aquatica (DA1877),... etc. Also, state the names in the same order between the text and figures. This would greatly reduce confusion.*
We agree this would be helpful and this will be altered as requested.

*Do you have suggestions that would help the authors improve the presentation of their data and conclusions?*
***1.*** *I would suggest bringing the focus and developing the logical arguments and presentation of the experiments around food sensing, behaviour and metabolism rather than DR-longevity, as it appears in the first figure that mediating DR-longevity is not the biological function of skn-1b, but rather a consequence of its role in feeding/food-sensing.*

We understand this comment and will integrate this viewpoint as we remodel the manuscript.

***2.*** *I found the explanation of daf-11 regulating skn-1b levels a little confusingly written. The exploration data implies an epistatic relationship, the expression pattern data (and what is known of daf-11 function) implies daf-11 drives the link between the external environment and SKN-1B expression levels.*

The data from the new, planned experiments will help clarify this section.

***3.*** *The inclusion of referencing supplementary figures within the figure legends, and including citations, was confusing to me. I would recommend including these references in the text as they are required, and thoroughly checking which supplementary figures are really required for inclusion to the manuscript. If the findings of the supplementary figures are not relevant to understanding/interpreting the main text I would suggest excluding them.*

As suggested, we will reference supplementary figures in the text (as well as legends where appropriate) to help avoid confusion. At present we do not envisage removing any of the figures but, as we address the comments of the reviewers and rewrite the manuscript this will be kept under review.

*Describe the nature and significance of the advance (e.g. conceptual, technical, clinical) for the field.*
*The ASI neurons are key drivers of food-related behaviour in the worm, and understanding the complexities (and subtleties) of the transcriptional and signalling networks that govern the functional output of these neurons is very interesting. This work adds an additional component to the significant body of work the exists about ASI function. Given that many of the observations and pathways described have been previously published, the nature of this work is less a significant conceptual advance, but rather an extension of our knowledge and understanding of how the ASI neurons can integrate signals from the external environment and propagate these signals, in part through skn-1b activity to the internal metabolic state of the animal.*
Our work identifies SKN-1B as a novel component of the neuroendrocrine network that underlies ASI control of behaviour and mitochondrial physiology. Identifying SKN-1B as a metabolic regulator in this context significantly expands and consolidates what we know of other reported behavioural regulators (*daf-11, daf-7, daf-2* and *daf-16*), forming a coherent picture of how food is sensed by the animal, information computed by the ASI neurons, and the appropriate behavioural and physiological responses instigated. In addition, SKN-1/Nrfs have not been described in these roles before, so this work opens up the novel idea that mammalian Nrfs could also be involved in food sensing and satiety related behaviour. Taken together, this is an important, conceptual, extension of our knowledge.

*Place the work in the context of the existing literature (provide references, where appropriate).*
*There is a large body of work that demonstrates the importance of the ASI neurons, TGF-beta and insulin-like signalling in feeding, metabolic regulation and food-related behaviour. This work places skn-1b as an environmentally-responsive transcription factor acting in the ASI neurons that modulates outputs of the ASI neurons.*

*Define your field of expertise with a few keywords to help the authors contextualize your point of view. Indicate if there are any parts of the paper that you do not have sufficient expertise to evaluate.*
*Feeding behaviour, neuronal transcriptional programs, metabolism.
Not a longevity expert or an electron microscopy expert.*

REFEREES CROSS-COMMENTING

The review of Reviewer 1 was inappropriate in my opinion. Very few manuscripts are that perfect and having no concerns or suggestions for improvement is worrying. The review system is obviously not there to hinder publication but to provide an unbiased view of the experimentation, data and conclusions drawn.

Reviewer 2 had similar concerns as myself. The manuscript is of interest however the suggested experiments and clarifications by both of us would improve the manuscript.
